# Supplementary material for: Ebola Virus Disease mathematical models and epidemiological parameters: a systematic review
Source: Lancet Infect Dis. Author manuscript; Available in PMC 2024 Dec 1. (PMC7616620; doi:10.1016/S1473-3099(24)00374-8)
Supplement: Supplementary Material [file EMS197979-supplement-Supplementary_Material.pdf]

Supplementary Material: Ebola Virus Disease mathematical models and epidemiological parameters: a systematic review and meta-analysis

Rebecca K. Nash[1*], Sangeeta Bhatia[1,2,3*], Christian Morgenstern[1], Patrick Doohan[1], David Jorgensen[1], Kelly McCain[1], Ruth McCabe[4,5], Dariya Nikitin[1], Alpha Forna[6], Gina Cuomo-Dannenburg[1], Joseph T. Hicks[1], Richard J. Sheppard[1], Tristan Naidoo[1], Sabine van Elsland[1], Cyril Geismar[1], Thomas Rawson[1], Sequoia Iris Leuba[1], Jack Wardle[1], Isobel Routledge[7], Keith Fraser[1], Pathogen Epidemiology Review Group[m], Natsuko Imai-Eaton[1], Anne Cori[1], and H. Juliette T. Unwin[8,1,c]

[1]MRC Centre for Global Infectious Disease Analysis & WHO Collaborating Centre for Infectious Disease Modelling, Jameel Institute, School of Public Health, Imperial College London, UK
[2]Health Protection Research Unit in Modelling and Health Economics
[3]Modelling and Economics Unit, UK Health Security Agency, London, UK
[4]Department of Statistics, University of Oxford, Oxford, UK
[5]Health Protection Research Unit in Emerging and Zoonotic Infections. University of Liverpool, Liverpool, UK
[6]Center for the Ecology of Infectious Diseases, Odum School of Ecology, University of Georgia, Athens, GA, USA
[7]Institute of Global Health Sciences, University of California, San Francisco, USA
[8]School of Mathematics, University of Bristol, Bristol, UK
[*]Equal contribution
[c]Corresponding author: Dr H. Juliette T. Unwin, 1.86 School of Maths, Fry Building, Univ. of Bristol, Woodland Road, Bristol, BS8 1UG, Juliette.unwin@bristol.ac.uk

Contents

1 Overview **4**

2 Ebola outbreaks **4**

3 Additional Methods **5**

3.1 Study Selection 5
3.2 Inclusion and exclusion criteria 5
3.3 Backwards citation screening 5
3.4 Data extraction 6
3.4.1 Article 6
3.4.2 Parameters 7
3.4.3 Model 8
3.5 Quality Assurance 8
3.6 Data analysis 9
3.7 PRISMA checklist 11

4 Results **14**

4.1 Overview of all EVD parameters 14
4.2 Seroprevalence 16
4.2.1 Seroprevalence in countries with history of officially reported EVD cases 16
4.2.2 Seroprevalence in countries with no history of officially reported EVD cases 17
4.2.3 Risk factors associated with seropositivity 18
4.3 Transmission parameters 19
4.3.1 Attack rate 19
4.3.2 Basic reproduction number 20
4.3.3 Effective reproduction number 24
4.3.4 Secondary attack rate 26
4.3.5 Growth rate 27
4.3.6 Doubling time 28
4.3.7 Overdispersion 29
4.3.8 Risk factors associated with transmission 31
4.4 Temporal dynamics of disease progression 32
4.4.1 Time periods for the infection process 32
4.4.2 Delays beyond symptom onset 33
4.4.3 Delays beyond admission to care 34
4.4.4 QA unfiltered meta-analysis by Ebola virus species 35
4.4.5 QA unfiltered meta-analysis 37
4.5 Severity 38
4.5.1 Case Fatality Ratio (CFR) 38
4.5.2 Risk factors associated with severity 43
4.6 Mutation rates 44
4.7 Transmission Models 45

5 Pathogen Epidemiology Review Group (PERG) membership **54**

List of Figures

S1 QA scores (%) across parameters. 9
S2 Time periods for the infection process 32
S3 All delays from symptom onset to various endpoints 33
S4 Delays beyond admission to care. 34
S5 Meta-analysis for A) incubation period B) onset-to-death and C) onset-to-recovery regardless of QA score, with a species sub-group analysis 36
S6 Meta-analysis for A) infectious period and B) serial interval regardless of QA score 37

List of Tables

S1 Outbreaks of Ebola Virus Disease 4
S2 Inclusion and exclusion criteria 6
S3 Review papers used in backwards citation screening 6
S4 Quality assessment questionnaire 8
S5 PRISMA 2020 Abstracts Checklist 11
S6 PRISMA 2020 Checklist. 12
S7 Overview of the total number of parameters extracted within each parameter group 14
S8 Overview of the total number of parameters extracted for each parameter type 15
S9 Seroprevalence in countries with history of officially reported EVD cases 16
S10 Seroprevalence in countries with no history of officially reported EVD cases and multi-country analyses 17
S11 Risk factors for seropositivity 18
S12 Attack rates 19
S13 Basic R estimates 21
S14 Effective R estimates 24
S15 Secondary attack rates 26
S16 Growth rates 27
S17 Doubling times 28
S18 Overdispersion table 30
S19 Risk factors for infection table 31
S20 Risk factors for onward transmission by significance and adjustment. 31
S21 Case fatality ratio results 39
S22 Risk factors associated with severity 43
S23 Mutation rates 44
S24 Summary of EVD model types. 45
S25 Summary of EVD model assumptions 45
S26 Models overview 46
S27 Summary of EVD model's with code 53
S28 Pathogen Epidemiology Review Group (PERG) membership 54

1 Overview

In this document, we present additional details and results from our systematic review of mathematical models and epidemiological parameters of Ebola Virus Disease. We first list the 38 known outbreaks of Ebola Virus in Section 2. We then present details of the search terms, inclusion and exclusion criteria, data extraction process, and quality assessment strategy (Section 3). Section 4.1 gives an overview of the epidemiological parameters extracted. Subsequent sections (Sections 4.2 to 4.6) detail results on specific parameters, including summaries across all studies. Transmission models identified in our review are described in Section 4.7.

2 Ebola outbreaks

Table S1: Outbreaks of Ebola Virus Disease up to January 2023. This table has been adapted from [1] * = laboratory-acquired infection, ** = imported infection, ± = now South Sudan, ⋄ = cases reported in Guinea, Liberia, Sierra Leone, Mali, Nigeria, Senegal, Italy, Spain, UK & USA.

Year	Country	Location	Species
1976	DRC	Mongala Province	Zaire
1976	South Sudan	Western and Central Equatoria State	Sudan
1976	United Kingdom*	Wiltshire, England	Sudan
1977	DRC	Sud-Ubangi Province	Zaire
1979	South Sudan	Western Equatoria State	Sudan
1994	Gabon	Ogooué-Ivindo Province	Zaire
1994	Côte d'Ivoire	Taï National Park	Tai Forest
1995	DRC	Kwilu Province	Zaire
1996	Russia*	Unknown	Zaire
1996(a)	Gabon	Ogooué-Ivindo Province	Zaire
1996(b)	Gabon	Ogooué-Ivindo Province	Zaire
1996	South Africa**	Johannesburg	Zaire
2000-2001	Uganda	Northern and Western regions	Sudan
2001-2002	Gabon & Republic of the Congo	La Zadié, Ivindo and Mpassa Districts (Gabon) and Cuvette-Ouest Department (Republic of the Congo)	Zaire
2003(a)	Republic of the Congo	Cuvette-Ouest Department	Zaire
2003(b)	Republic of the Congo	Cuvette-Ouest Department	Zaire
2004	Sudan±	Western Equatoria State	Sudan
2004	Russia*	Unknown	Zaire
2005	DRC	Cuvette-Ouest Department	Zaire
2007	DRC	Kasai (formerly Kasaï-Occidental)	Zaire
2007	Uganda	Western Region	Bundibugyo
2008-2009	DRC	Kasai (formerly Kasaï-Occidental)	Zaire
2011	Uganda	Central Region	Sudan
2012	Uganda	Western Region	Sudan
2012	DRC	Haut-Uélé Province (formerly Orientale)	Bundibugyo
2012-2013	Uganda	Central Region	Sudan

2013-2016	West-Africa$^{\diamond}$		Zaire
2014(b)	DRC	Équateur Province	Zaire
2017	DRC	Bas-Uélé Province	Zaire
2018	DRC	Équateur Province	Zaire
2018-2020	DRC (& Uganda)	North Kivu, Ituri and South Kivu Provinces	Zaire
2020	DRC	Équateur Province	Zaire
2021(a)	DRC	North Kivu Province	Zaire
2021	Guinea	Nzérékoré Region	Zaire
2021(b)	DRC	North Kivu Province	Zaire
2022(a)	DRC	Équateur Province	Zaire
2022(b)	DRC	North Kivu Province	Zaire
2022-2023	Uganda	Central, Eastern and Western regions	Sudan

3 Additional Methods

3.1 Study Selection

We searched PubMed and Web of Science initially on 8th March 2019 and then updated our search on 7th July 2023 using the following search terms: Ebola AND ((transmissi* OR epidemiolog*) OR (model* NOT imag*) OR (severity OR "case fatality ratio*" OR CFR OR "case fatality rate*" OR "mortality rate*" OR "attack rate*") OR ("infectious period*" OR "serial interval*" OR "incubation period*" OR "generation time*" OR "generation interval*" OR "latent period*" OR latency) OR (heterogeneit* OR superspread* OR "super spread*" OR super-spread* OR overdispersion OR overdispersed OR over-dispersion OR over-dispersed OR "over dispersion" OR "over dispersed") OR (infectivity OR infectiousness OR "growth rate*" OR "reproduction number*" OR "reproductive number*" OR R0 OR "reproduction ratio*" OR "reproductive rate*") OR ("pre-existing immunity" OR serological OR serology OR serosurvey*) OR (evolution* OR mutation* OR substitution*) OR (outbreak* OR cluster* OR epidemic*) OR ("risk factor*")).

We used the online platform Covidence to perform title screening, abstract screening, and full text review. Our inclusion and exclusion criteria are listed in full in Table S2. Each paper was screened by two reviewers, with disagreements resolved by consensus. No reason was recorded for exclusion at the title and abstract stage. At the full text screening stage, a reason for exclusion was selected from the exclusion criteria.

3.2 Inclusion and exclusion criteria

Following our PROSPERO registration CRD42023393345, our inclusion / exclusion criteria are given in SM Table S2. We did not extract outbreak size information since the Ebola outbreaks are well documented (see Table S1 for a full list [1]). We excluded correspondence, letters to editors with the exception of Nature Letters, which published short articles reporting primary research and conference proceedings, unless they were known to be peer-reviewed.

We excluded reviews at the full-text extraction stage but checked the cited literature to ensure that we had included all relevant papers.

3.3 Backwards citation screening

We identified 179 reviews during our screening process and chose to check we had included all the references from the 11 papers with systematic or meta-analysis in their title that were on relevant topics. We also included one additional review [2] which we knew was highly cited for modelling parameters.

Table S2: Inclusion and exclusion criteria

**Inclusion**	**Exclusion**
Measures/estimates of human: Reproduction numbers (R, R_0, R_t, r, R_e), growth rate (r), doubling times, generation time, serial interval, incubation/latent period, case fatality ratio (CFR), attack rate, mutation rate (e.g. from phylogenetic study), overdispersion, risk factors (risk and the measure).	Non-English language publication
Mathematical or statistical model of transmission.	Studies of co-infections. (local, regional, national, international).
Measures of seroprevalence and negative seroprevalence in humans.	Animal studies.
Nature letters.	Qualitative studies, e.g., KAP studies.
	Pathogen not the primary focus of study.
	Duplicates.
	Does not match any of the inclusion criteria.
	In-vitro studies.
	Non-peer reviewed publications, conference proceedings, abstracts, posters, letters to the editor

Table S3: Review papers used in backwards citation screening

Parameter	Reviews
Case fatality ratios	VanKerkhove2015 [2], Rojek2019 [3], Kawuki2021 [4], Nyakarahuka2016 [5], Belhadi2022 [6]
Risk factors	VanKerkhove2015 [2], Brainard2016 [7], Selveraj2018 [8]
Incubation period	VanKerkhove2015 [2], Velasquez 2015 [9]
Latent period	Velasquez2015 [9]
Serial interval	VanKerkhove2015 [2]
Other delays	VanKerkhove2015 [2]
Reproduction number	VanKerkhove2015 [2]
Secondary attack rates	Dean2016 [10]
Seroprevalence	Bower2017 [11], Nyakarahuka2016 [5] and Rojek2019 [3]
Modelling reviews	Wong2017 [12], Abdalla2022 [13]

3.4 Data extraction

We extracted the data using a bespoke Microsoft Access (Version 2305) database. The extractors extracted data using three forms that then populated the data tables. The exact questions asked in the form and full list of parameters extracted can be found in the Wiki of out GitHub repository for our package (https://github.com/mrc-ide/epireview).

3.4.1 Article

We extracted meta information about the paper including the first author, title, journal, digital object identifier (DOI) and page numbers where available to identify the papers. Each article was scored using seven questions about the quality or risk of bias of the paper (see SM Section 3.5).

3.4.2 Parameters

Our extraction focused on parameters related to seroprevalence, transmission, natural history, severity and evolution. We extracted the following information for each category:

- Seroprevalence
  - seroprevalence in countries with and without reported outbreaks,
  - risk factors for seropositivity.
- Transmission
  - the basic and effective reproduction numbers,
  - growth rate,
  - doubling time,
  - attack rate,
  - secondary attack rate (defined as the probability that an infection occurs among susceptible people within a specific group for example household),
  - overdispersion (defined as overdispersion in the offspring distribution) and
  - risk factors associated for transmission.
- Natural history
  - time periods for the infection process including the serial interval (defined as time between symptoms onset in the infector to symptoms onset in the infectee), generation time (defined as time between infection in the infector and infectee) and incubation period (defined as the time between infection and symptoms onset in an individual),
  - delays beyond symptom onset and
  - delays beyond admission to care.
- Severity
  - case fatality ratios (CFRs) and
  - risk factors associated with severity.
- Evolution
  - evolutionary rates,
  - mutation rates and
  - substitution rates.

We extracted the parameter value type (e.g., mean, standard deviation, median) and uncertainty measure (capturing the precision of estimates) for each parameter when available. If more than three estimates for a single parameter type were presented in a paper, e.g. from different populations, using different methods, or across different time periods, these estimates were extracted as a range of central estimates. Contextual information about the study location and dates, sample size, basic demographic information, and timing of the survey in relation to reported outbreaks were also extracted.

We recorded the methods used for reproduction number estimation (e.g. renewal equations, empirical methods or next generation matrices). For CFRs, we extracted whether the estimation approach was naive or adjusted and recorded numerators and denominators for the CFR and seroprevalence estimates where available. For genomic data, we noted the gene studied if specified and whether the sequence data were available.

We did not extract odds ratio estimates for risk factors because it would be difficult to compare them due to differences in stratifications or reference groups. Instead, we chose to extract an overview of the risk factors explored across studies. This included the outcome of interest e.g. death, the risk factor for that outcome e.g. sex, whether the estimates were statistically significant and whether they were from univariate or multivariate analyses.

3.4.3 Model

We extracted details of Ebola transmission models that were either fit to data or theoretical only. This included the type of model, transmission routes, whether the model was deterministic or stochastic, assumptions, interventions, the availability of model code, and in the case of a compartmental model, model subclassification (e.g., SIR, SEIR).

We extracted further information from the 37 models that had publicly available code. This included the purpose of the model, health states considered, if uncertainty was included in the model, if the model was fit and the method, the type of data used, the period of the outbreak modelled, if the model was spatial, if it included spillover, what interventions were considered, the language the model was written in, a link to the code and if there were instructions on how to use it.

3.5 Quality Assurance (QA)

Given the diversity of papers included in our review, we found that existing quality assessment criteria did not align with our research. We therefore designed a bespoke questionnaire consisting of seven questions SM Table S4 to assess the quality of each article. Further information about how these questions were interpreted for each parameter can be found in the Wiki of our GitHub repository for our package (https://github.com/mrc-ide/epireview).

Table S4: Quality assessment questionnaire

Theme	Question
Is the methodological/statistical approach suitable? (how the data are used)	1. Clear and reproducible
	2. Robust and appropriate for the aim [subjective criteria]
Are the assumptions appropriate? (input parameters/assumptions - what goes into the methodology)	3. Clear and reproducible
	4. Justified (published study or analysis of data)[objective criteria]
Are the data appropriate for the selected methodological approach?	5. Clearly described and reproducible
	6. Are issues in the data clearly discussed and acknowledged?
	7. Are issues in the data accounted for in the chosen methodological approach?

We observed considerable variability in the QA scores across the extracted parameters (Figure S1). Among 11 included articles published before 1991, only one had a QA score surpassing 50%. Among the 43 included articles published between 1991 and 2014, no discernible trend in QA scores was observed. However, a surge in articles related to EVD emerged after 2014, coinciding with the West African Ebola Epidemic. Of the 522 articles included in our review, 468 were published after 2014. 36% of these articles had a QA score below 50%.

Given the volume of and variability in the data, we aimed to focus on estimates from high quality articles. Therefore, in the figures and tables presented in the main text, we only included parameters from articles with a QA score of at least 50% (which encompasses just below three-quarters (73%) of all articles). The threshold value was selected to balance the inclusion of as much data as possible with the robustness of our analysis, aiming to minimise the influence of unreliable estimates on the results.

A sensitivity analysis for the meta-analyses showed that the inclusion of parameters from low QA scoring papers produced similar results. All parameters are included in the tables and figures in the Supplementary Material.

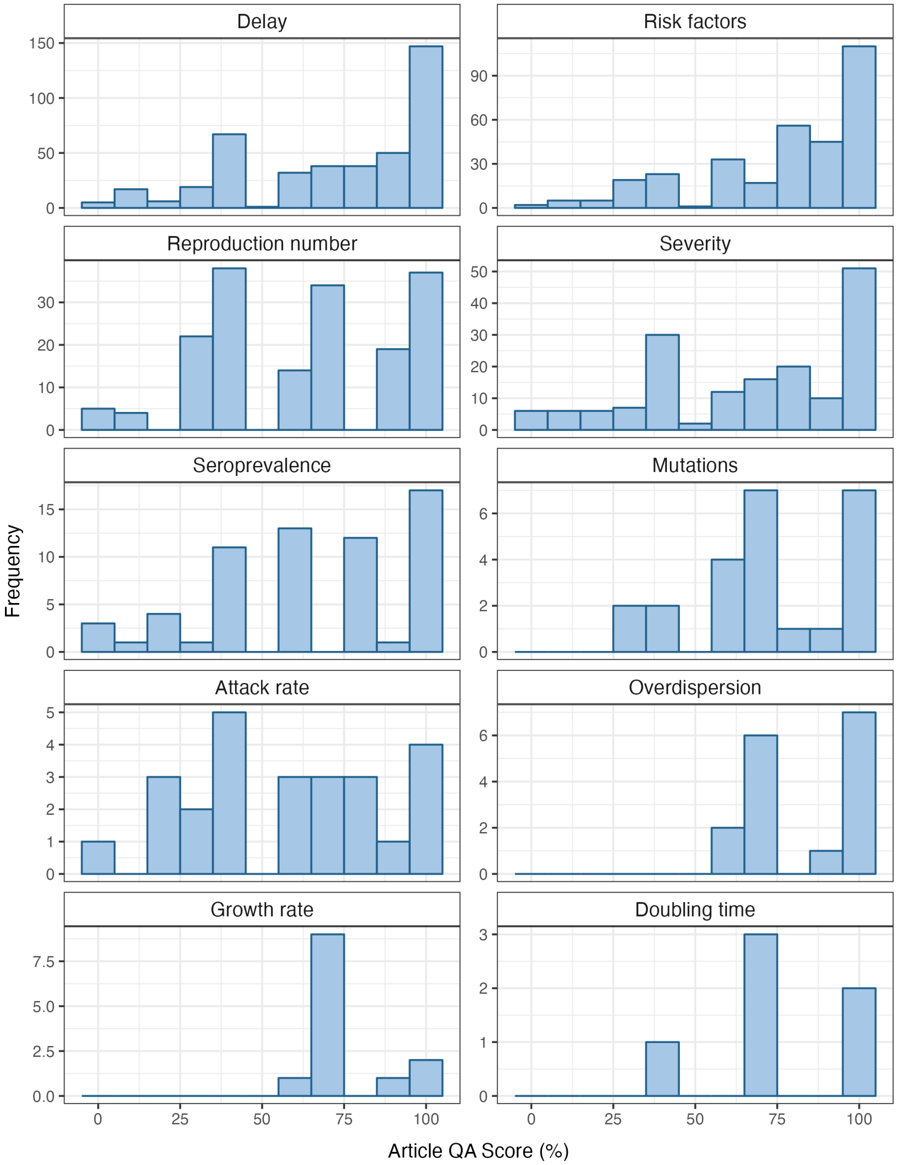

Figure S1: QA scores (%) across parameters.

3.6 Data analysis

All analysis was performed in R using orderly2 for workflow management (https://github.com/mrc-ide/priority-pathogens) and epireview for review specific functions and data storage.

We did not extract information on EVD outbreaks as they are well documented; instead we assigned an outbreak to each extracted parameter based on the country and time for which the parameter had been estimated.

For meta-analyses, we used the metamean function from the meta R package [14]. Parameters were only included in meta-analyses if studies reported a sample size alongside paired (i) parameter means with a standard deviation (SD) of the sample, (ii) means with standard errors, or (iii) medians with a range or interquartile range. For (ii) we converted the standard error to SD using the sample size [15]. For (iii) we used the functionality of metamean to derive the mean and SD using the method outlined by Cai et al. [16], which allows for unknown non-normal

distributions. Where data for multiple EV species were available in this format, we used metamean to perform meta-analyses with species sub-groups with p-values being calculated using a χ^2 test.

For CFR, we chose not to perform a meta-analysis because the study estimates were very context specific. However, we now provide naive syntheses of the heterogeneous estimates, calculated as an unweighted and weighted mean (weighted by the sample size), with recommendation to use the weighted mean

3.7 PRISMA checklist

Table S5: PRISMA 2020 Abstracts Checklist

Section & Topic	Item #	Checklist item	Reported (Yes/No)
**Title**			
Title	1	Identify the report as a systematic review.	Yes
**Background**			
Objectives	2	Provide an explicit statement of the main objective(s) or question(s) the review addresses.	Yes
**Methods**			
Eligibility criteria	3	Specify the inclusion and exclusion criteria for the review.	Yes
Information sources	4	Specify the information sources (e.g. databases, registers) used to identify studies and the date when each was last searched.	Yes
Risk of bias	5	Specify the methods used to assess risk of bias in the included studies.	Yes
Synthesis of results	6	Specify the methods used to present and synthesise results.	Yes
**Results**			
Included studies	7	Give the total number of included studies and participants and summarise relevant characteristics of studies.	Yes
Synthesis of results	8	Present results for main outcomes, preferably indicating the number of included studies and participants for each. If meta-analysis was done, report the summary estimate and confidence/credible interval. If comparing groups, indicate the direction of the effect (i.e. which group is favoured).	Yes
**Discussion**			
Limitations of evidence	9	Provide a brief summary of the limitations of the evidence included in the review (e.g. study risk of bias, inconsistency and imprecision).	Yes
Interpretation	10	Provide a general interpretation of the results and important implications.	Yes
**Other**			
Funding	11	Specify the primary source of funding for the review.	Yes
Registration	12	Provide the register name and registration number.	Yes

Table S6: PRISMA 2020 Checklist.

**Section & Topic**	**Item #**	**Checklist item**	**Location reported**
**Title**			
Title	1	Identify the report as a systematic review.	page 1
**Abstract**			
Abstract	2	See the PRISMA 2020 for Abstracts checklist.	Table S5
**Introduction**			
Rationale	3	Describe the rationale for the review in the context of existing knowledge.	page 2
Objectives	4	Provide an explicit statement of the objective(s) or question(s) the review addresses.	page 5
**Methods**			
Eligibility criteria	5	Specify the inclusion and exclusion criteria for the review and how studies were grouped for the syntheses.	page 5
Information sources	6	Specify all databases, registers, websites, organisations, reference lists and other sources searched or consulted to identify studies. Specify the date when each source was last searched or consulted.	page 5
Search strategy	7	Present the full search strategies for all databases, registers and websites, including any filters and limits used.	page 5 + Figure 1
Selection process	8	Specify the methods used to decide whether a study met the inclusion criteria of the review, including how many reviewers screened each record and each report retrieved, whether they worked independently, and if applicable, details of automation tools used in the process.	page 5
Data collection process	9	Specify the methods used to collect data from reports, including how many reviewers collected data from each report, whether they worked independently, any processes for obtaining or confirming data from study investigators, and if applicable, details of automation tools used in the process.	page 5
Data items	10a	List and define all outcomes for which data were sought. Specify whether all results that were compatible with each outcome domain in each study were sought (e.g. for all measures, time points, analyses), and if not, the methods used to decide which results to collect.	page 6, SM Section 3.4.2
	10b	List and define all other variables for which data were sought (e.g. participant and intervention characteristics, funding sources). Describe any assumptions made about any missing or unclear information.	page 6, SM Section 3.4.2
Study risk of bias assessment	11	Specify the methods used to assess risk of bias in the included studies, including details of the tool(s) used, how many reviewers assessed each study and whether they worked independently, and if applicable, details of automation tools used in the process.	page 5
Effect measures	12	Specify for each outcome the effect measure(s) (e.g. risk ratio, mean difference) used in the synthesis or presentation of results.	page 6
Synthesis methods	13a	Describe the processes used to decide which studies were eligible for each synthesis (e.g. tabulating the study intervention characteristics and comparing against the planned groups for each synthesis (item #5)).	page 6
	13b	Describe any methods required to prepare the data for presentation or synthesis, such as handling of missing summary statistics, or data conversions.	page 6
	13c	Describe any methods used to tabulate or visually display results of individual studies and syntheses.	page 6
	13d	Describe any methods used to synthesize results and provide a rationale for the choice(s). If meta-analysis was performed, describe the model(s), method(s) to identify the presence and extent of statistical heterogeneity, and software package(s) used.	page 6
	13e	Describe any methods used to explore possible causes of heterogeneity among study results (e.g. subgroup analysis, meta-regression).	page 6
	13f	Describe any sensitivity analyses conducted to assess robustness of the synthesized results.	-
Reporting bias assessment	14	Describe any methods used to assess risk of bias due to missing results in a synthesis (arising from reporting biases).	page 5
Certainty assessment	15	Describe any methods used to assess certainty (or confidence) in the body of evidence for an outcome.	page 5
**Results**			

Study selection	16a	Describe the results of the search and selection process, from the number of records identified in the search to the number of studies included in the review, ideally using a flow diagram.	Figure 1
	16b	Cite studies that might appear to meet the inclusion criteria, but which were excluded, and explain why they were excluded.	Available on request
Study characteristics	17	Cite each included study and present its characteristics.	SM Two
Risk of bias in studies	18	Present assessments of risk of bias for each included study.	SM Section 3.5
Results of individual studies	19	For all outcomes, present, for each study: (a) summary statistics for each group (where appropriate) and (b) an effect estimate and its precision (e.g. confidence/credible interval), ideally using structured tables or plots.	pages 6-10
Results of syntheses	20a	For each synthesis, briefly summarise the characteristics and risk of bias among contributing studies.	SM
	20b	Present results of all statistical syntheses conducted. If meta-analysis was done, present for each the summary estimate and its precision (e.g. confidence/credible interval) and measures of statistical heterogeneity. If comparing groups, describe the direction of the effect.	page 8/9
	20c	Present results of all investigations of possible causes of heterogeneity among study results.	page 6-10
	20d	Present results of all sensitivity analyses conducted to assess the robustness of the synthesized results.	Figure S5
Reporting biases	21	Present assessments of risk of bias due to missing results (arising from reporting biases) for each synthesis assessed.	Figure S5
Certainty of evidence	22	Present assessments of certainty (or confidence) in the body of evidence for each outcome assessed.	pages 6-10
**Discussion**			
Discussion	23a	Provide a general interpretation of the results in the context of other evidence.	page 20
	23b	Discuss any limitations of the evidence included in the review.	page 21
	23c	Discuss any limitations of the review processes used.	page 21
	23d	Discuss implications of the results for practice, policy, and future research.	page 21-22
**Other Information**			
Registration and protocol	24a	Provide registration information for the review, including register name and registration number, or state that the review was not registered.	page 1
	24b	Indicate where the review protocol can be accessed, or state that a protocol was not prepared.	page 1
	24c	Describe and explain any amendments to information provided at registration or in the protocol.	n/a
Support	25	Describe sources of financial or non-financial support for the review, and the role of the funders or sponsors in the review.	page 2
Competing interests	26	Declare any competing interests of review authors.	page 22
Availability of data, code and other materials	27	Report which of the following are publicly available and where they can be found: template data collection forms; data extracted from included studies; data used for all analyses; analytic code; any other materials used in the review.	page 22

4 Results

4.1 Overview of all EVD parameters

Table S7: Overview of the total number of parameters extracted within each parameter group (we do not include parameters in this table where data is available only in the figures).

Parameter Group	Total Parameters
Delay	420
Risk factors	316
Reproduction number	173
Severity	166
Seroprevalence	63
Attack rate	25
Mutations	24
Overdispersion	16
Growth rate	13
Doubling time	6

Table S8: Overview of the total number of parameters extracted for each parameter type. * = "Other delay" encompasses the 32 other delay types that were only extracted from a single study.

Parameter Type	Total Parameters
**Delay**	
Symptom onset to admission to care	60
Incubation period	52
Symptom onset to death	48
Admission to care to death	33
Other delay*	33
Infectious period	27
Admission to care to death/discharge	22
Serial interval	22
Admission to care to discharge from care	20
Symptom onset to recovery/non-infectiousness	15
Symptom onset to reporting	15
Symptom onset to discharge from care	14
Admission to care to recovery/non-infectiousness	12
Latent period	11
Death to burial	7
Symptom onset to quarantine	5
Symptom onset to seeking care	4
Symptom onset to test	4
Reporting to death	3
Reporting to discharge from care	3
Exposure/infection to admission to care	2
Exposure/infection to death	2
Funeral start to funeral end	2
Symptom onset to antibody detection (IgM/IgG)	2
Symptom onset to other	2
**Risk factors**	
Risk factors	316
**Reproduction number**	
Basic reproduction number (R0)	118
Effective reproduction number (Re)	55
**Severity**	
Case Fatality Ratio (CFR)	166
**Seroprevalence**	
IgG	36
IFA	13
Unspecified	8
IgM	6
**Mutations**	
Evolutionary rate	12
Substitution rate	10
Mutation rate	2
**Attack rate**	
Secondary attack rate	15
Attack rate	10
**Overdispersion**	
Overdispersion	16
**Growth rate**	
Growth rate (r)	13
**Doubling time**	
Doubling time	6

4.2 Seroprevalence

4.2.1 Seroprevalence in countries with history of officially reported EVD cases

Table S9: Seroprevalence in countries with history of officially reported EVD cases. * = These are not duplicate entries. One entry corresponds to Zaire antigens, and the other corresponds to Sudan antigens.

Country	Location	Survey date	Central estimate	Central range	Central type	Uncertainty (95% CI)	Population Sample	Sample size	Disaggregated by	Article	QA score (%)
**Seroprevalence - IFA**											
DRC	Bumba Zone	1 Sep 1977 - 24 Oct 1977					Community based		Age, Other, Sex, Symptoms	International Commission 1978	40.0
DRC	Sud-Ubangi	1981 - 1985	15.00				Contact based	188	Age	Jezek 1999	20.0
DRC	Mabalako, Beni, Butembo, Katwa	Unspecified	2.30			1.1 - 4	Hospital based	488	Other	Nkuba-Ndaye 2022	80.0
Gabon	Makokou	1994 - Unspecified	0.00				Hospital based			Georges 1999	80.0
Guinea		12 May 2016 - 8 Sep 2017	4.10	3.32 - 20		3.12 - 5.28	Contact based	1,390	Level of exposure, Other, Sex, Symptoms	Diallo 2019	100.0
Sudan	Nzara Maridi	Jun 1976 - Nov 1976		19 - 83			Other		Occupation, Other, Region	WHO/Int. Study Team 1978	60.0
Uganda	Karamoja	May 1984	3.00				Community based	132	Region	Rodhain 1989*	20.0
Uganda	Karamoja	May 1984	3.00				Community based	132	Region	Rodhain 1989*	20.0
**Seroprevalence - IgG**											
DRC	Kikwit	May 1995 - Jul 1995	9.00	4 - 31			Hospital based	429	Occupation	Tomori 1999	60.0
DRC	Kikwit	28 Jul 1995 - 16 Aug 1995	2.20	0 - 11.1			Trade / business based	414	Occupation	Busico 1999	60.0
DRC	Kikwit	18 Aug 1995 - 6 Sep 1995	9.30	0 - 18.1	Other		Community based	161	Age, Occupation, Other, Region, Sex	Busico 1999	60.0
DRC	Watsa region	Aug 2002	18.70		Mean	14.4 - 23.5	Community based	300	Sex	Mulangu 2016	80.0
DRC	Sankuru district	Aug 2007 - Sep 2007	11.00				Community based	3,415		Mulangu 2018	80.0
DRC	Boende, Kabondo-Dianda, Yambuku, North and South Kikwit	Sep 2015 - Aug 2017	8.30		Other		Community based	1,366		Bratcher 2021	100.0
DRC	Boende health zone	Sep 2015 - Nov 2015	28.10				Hospital based	565		Hoff 2019	60.0
DRC	Boende health zone	Sep 2015 - Nov 2015	15.80				Hospital based	565		Hoff 2019	60.0
DRC	Boende	Nov 2015	22.50	0 - 37			Hospital based	582	Age, Level of exposure, Other, Sex	Doshi 2022	80.0
DRC	North Kivu Province, Rubare, Kiwanja Umoja, Biruma, Ntamugemga, Kalengera	May 2017 - Apr 2018	11.03				Household based	272	Region	Goldstein 2020	57.1
DRC	Mbandaka	Jun 2018 - Jul 2018	4.60				Hospital based	539	Other	Shaffer 2022	80.0
DRC	Mbandaka, Equateur Province	12 Nov 2018 - 16 Nov 2018	5.00		Other		Trade / business based	19		Lucas 2020	40.0
Gabon	Lastoursville, Makokou, Matadi-Ngouassa, Moukoro, Doussleoussou, Doussala	1981 - 1997	1.20	0 - 2.2	Other		Community based	1,147	Region, Sex	Lahm 2007	100.0
Gabon		24 Jan 1996 - 4 Feb 1996	10.20		Mean		Community based	235	Age, Occupation, Sex	Bertherat 1999	40.0
Gabon	Mayibout, Mvadi villages	Feb 1996 - Unspecified		14.9 - 30			Hospital based	205	Region	Georges 1999	80.0
Gabon	Ogooue-Ivindo region	Jul 1997 - Oct 1997	1.00	1 - 1.4	Mean	0.5 - 1.9	Community based	2,533		Heffernan 2005	100.0
Gabon	Rural Gabonese populations	Jun 2005 - Sep 2008	15.30	1.5 - 32.4		14.3 - 16.5	Community based	4,349	Region	Nkoghe 2011	60.0
Gabon		2005 - 2008	15.30				Population based	4,349	Age, Other, Region, Sex	Becquart 2010	100.0
Guinea		Jun 2012 - Oct 2012	0.07				Population based	1,483		Keita 2018	100.0
Guinea		23 Mar 2015 - 11 Jul 2016		59.41 - 99.8			Hospital based		Other, Time	Diallo 2021	100.0
Guinea, Liberia, Sierra Leone	Lassa Diagnostic Laboratory	2007 - 2014	5.20	0 - 10			Other	672	Time	O'Hearn 2016	40.0
Republic of the Congo	Brazzaville, Pointe-Noire, Cuvette-Ouest	Mar 2011 - Jul 2011	2.50	1.6 - 4			Community based	809	Region	Moyen 2015	100.0
Sierra Leone	Bombali District	Feb 2015 - Mar 2015		3.8 - 100			Other		Other, Region	Mafopa 2017	60.0
Sierra Leone		Mar 2015 - Dec 2015		21.2 - 27.9			Hospital based	694		Liu 2018	100.0
Sierra Leone		3 Jul 2015 - 10 Sep 2015		0 - 100		0 - 100	Household based	486	Age, Occupation, Other, Sex, Symptoms	Glynn 2017	100.0
Sierra Leone	Sukudu	Oct 2015 - Jan 2016	22.00				Housing estate based	221		Kelly 2018	85.7
Sierra Leone	Kambia District	16 Mar 2016 - 29 Jun 2018	8.40	0 - 27.8	Other	7 - 10	Community based	1,282	Level of exposure, Occupation, Other	Manno 2022	100.0
Sierra Leone	Kono District	Sep 2016 - Jul 2017	9.50				Household based	421		Kelly 2022	40.0
Sierra Leone	Makeni	Mar 2017		40.1 - 97.7	Other		Other	481	Other	Halfmann 2019	40.0
Uganda	Ibanda, Kamwenge, Luweero	Jan 2015 - Feb 2015	2.30				Community based	724	Level of exposure	Nyakarahuka 2020	100.0
**Seroprevalence - IgM**											
DRC	Kikwit	28 Jul 1995 - 16 Aug 1995	0.00		Other		Trade / business based	414		Busico 1999	60.0
Gabon	Minkebe	27 Dec 1994	27.00				Unspecified	33		Amblard 1997	20.0
Gabon	Andock, Minkebe, Mekouka, Mvadi, Mayibout villages	Dec 1994 - Unspecified		1.8 - 10			Hospital based		Region, Time	Georges 1999	80.0
Sierra Leone		Mar 2015 - Dec 2015		2.4 - 11			Hospital based	694		Liu 2018	100.0
**Seroprevalence - Unspecified**											
DRC	Tandala	1978	7.00	1 - 21			Population based	1,096	Age, Region	Heymann 1980	28.6
DRC	Boende health zone	Sep 2015 - Nov 2015	9.50				Hospital based	565		Hoff 2019	60.0
DRC	Boende health zone	Sep 2015 - Nov 2015	2.80				Hospital based	565		Hoff 2019	60.0
Liberia	Bong County	15 Sep 2014 - 4 Jan 2015	42.00				Hospital based	382	Age, Occupation, Other, Region, Sex	Levine 2015	100.0
Sierra Leone	Sukundu	Dec 2014 - Jan 2015	7.50				Contact based	187		Richardson 2016	80.0
South Sudan	Nzara, Yambio	22 Sep 1979 - 9 Oct 1979	18.00	13 - 38			Household based	106	Level of exposure	Baron 1983	14.3

4.2.2 Seroprevalence in countries with no history of officially reported EVD cases

Table S10: Seroprevalence in countries with no history of officially reported EVD cases and multi-country analyses

Country	Location	Survey date	Central estimate	Central range	Central type	Uncertainty (95% CI)	Population Sample	Sample size	Disaggregated by	Article	QA score (%)
**Seroprevalence - IFA**											
Cameroon	Moloundou, Lomie, Lolodorf-Bipindi, Pete, Yaounde	1979 - 1980	9.70	3.2 - 23.5	Mean		Community based	1,517	Age, Other, Region	Bouree 1983	40.0
Central African Republic	Nola, Ikaumba, Bozo, Bangassou, Bouar, Obo, Mbre, Birao	Unspecified	21.30				Population based	4,295	Region	Johnson 1993	60.0
Djibouti	Randa, Djibouti City	1987	0.00				Other	160		Salah 1988	0.0
Kenya	Nzoia, Laisamis, Masinga, Lodwar, Malinda, Kilifi	Unspecified	1.42				Population based	1,899	Region	Johnson 1983	40.0
Multi-country: Africa (n = 6)	Cameroon, Central African Republic, Chad, Republic of the Congo, Equatorial Guinea, Gabon	Jan 1985 - Jun 1987	12.40	1.85 - 32.72	Other		Hospital based	5,070	Region	Gonzalez 1989	42.9
**Seroprevalence - IgG**											
Central African Republic	Lobaye district, Belemboke, Nola and Bangassou	Dec 1992 - Nov 1995	5.30	1.9 - 16.6			Community based	1,331	Region, Sex, Time	Paul Gonzalez 2000	40.0
Mali	Bamba, Banzana, Soromba	2015		1.5 - 6.1	Other		Other	600	Other	Bane 2021	40.0
Multi-country: Africa (n = 5)	Cameroon, DRC, Republic of the Congo, Ghana, Uganda	1997 - 2012	2.20	0 - 4.4		0.3 - 4.8	Other	2,430	Region	Steffan 2019	100.0
Tanzania		Jun 2018 - Nov 2018	1.80				Other	500	Age, Other, Region, Sex, Symptoms	Rugarabamu 2021	80.0
United Kingdom		16 Dec 2015 - 16 Jun 2016	0.75				Hospital based	268		Houlihan 2017	100.0
**Seroprevalence - IgM**											
Tanzania		Jun 2018 - Nov 2018	1.60				Other	500	Age, Other, Region, Sex, Symptoms	Rugarabamu 2021	80.0
Tanzania		Jun 2018 - Nov 2018	0.90				Hospital based	308	Age, Region, Sex	Rugarabamu 2022	80.0
**Seroprevalence - Unspecified**											
Madagascar	Antananarivo, Mandoto, Andasibe, Tsiroanomandidy, Ampijoroa	Unspecified	4.50				Population based	381	Region	Mathiot 1989	0.0
Madagascar	Antananarivo, Mandoto, Andasibe, Tsiroanomandidy, Ampijoroa	Unspecified	0.00				Population based	381	Region	Mathiot 1989	0.0

4.2.3 Risk factors associated with seropositivity

Table S11: Risk factors for seropositivity. Total is the number of parameters extracted for each risk factor for seropositivity.

Risk Factor for Seropositivity	Significant			Not significant			Total
	Adjusted	Not adjusted	Unspecified	Adjusted	Not adjusted	Unspecified	
Age	2	2	4	3	7	2	20
Sex	2	4	2	3	5	3	19
Occupation	1	2	2	1	7	0	13
Contact with animal	3	2	1	3	2	0	11
Close contact	1	2	0	1	4	0	8
Household contact	1	1	0	1	1	1	5
Funeral	1	1	0	1	1	0	4
Non-household contact	0	0	0	0	1	0	1
Other	6	3	4	5	8	1	27

4.3 Transmission parameters

4.3.1 Attack rate

Table S12: Attack rates

Country	Survey date	Central estimate (%)	Central range	Uncertainty (95% CI)	Population Sample	Sample size	Population Group	Disaggregated by	Article	QA score (%)
**South Sudan, 1976**										
Sudan	Jun 1976 - Nov 1976		0.34 - 1.42		Community based	280	Persons under investigation	Region	WHO/Int. Study Team 1978	60.0
**DRC, 1995**										
DRC	May 1995 - Jul 1995	9	4 - 31		Hospital based	429	Healthcare workers	Occupation	Tomori 1991	100.0
**Uganda, 2000-2001**										
Uganda	2000		0.01 - 0.238		Community based		Persons under investigation	Region	Okware 2015	20.0
**Republic of the Congo, 2005**										
Republic of the Congo	18 Apr 2005 - 8 Jul 2005	7.1			Community based		General population	Region	Nkoghe 2011 (b)	42.9
Republic of the Congo	18 Apr 2005 - 8 Jul 2005	8.6			Community based		General population	Region	Nkoghe 2011 (b)	42.9
Republic of the Congo	18 Apr 2005 - 8 Jul 2005	8.7			Community based		General population	Region	Nkoghe 2011 (b)	42.9
**Uganda, 2007**										
Uganda	29 Nov 2007 - 20 Feb 2008	0.043	0.003 - 0.185		Community based	116	General population	Age, Region, Sex	Wamala 2010	80.0
**West Africa 2013-2016**										
Guinea	22 Jun 2017 - 9 Jul 2017	7.29		4.38 - 11.28	Household based	247	General population		Timothy 2019	100.0
Liberia	14 May 2014 - 7 Mar 2015	0.25		0.15 - 0.4	Community based	17	General population		Kuehne 2016	28.6
Sierra Leone	May 2014 - Apr 2015	2.7			Household based	1161	General population		Caleo 2018	100.0

4.3.2 Basic reproduction number

Table S13: Basic R estimates

Country	Survey date	Central estimate	Central range	Central type	Uncertainty	Uncertainty type	Method	Disaggregated by	Article	QA score (%)
**DRC, 1976**										
DRC	Aug 1976 - Nov 1976	4.71	1.34 - 4.71	Median	3.92 - 5.66	95% CI	Compartmental model	Other	Camacho 2014	71.4
**DRC, 1995**										
DRC	1 Mar 1995 - 16 Jul 1995	1.40		Mean			Compartmental model		Lekone 2006	100.0
DRC	1 Mar 1995 - 12 Jul 1995	1.80		Unspecified			Compartmental model		Zaman 2009	100.0
DRC	1 Mar 1995 - 21 Jul 1995	2.11		Unspecified			Compartmental model		Ndanguza 2013	42.9
DRC	1 Mar 1995 - 21 Jul 1995	2.22		Mean	2.22 - 2.22	95% CrI			Ndanguza 2013	42.9
DRC	1 Mar 1995 - 21 Jul 1995	2.22		Mean	0.13	Standard Deviation	Compartmental model		Ndanguza 2013	42.9
DRC	6 Mar 1995 - 12 Jul 1995	1.83		Median	0.06	Standard Deviation	Compartmental model		Chowell 2004	28.6
DRC	Mar 1995 - Jul 1995		1.5 - 2.08	Mean			Compartmental model	Other	Ward 2023	42.9
DRC	1995	1.93		Median	1.74 - 2.78	IQR	Other		White 2007	85.7
DRC	1995	2.70		Mean	1.9 - 2.8	95% CI	Compartmental model		Legrand 2007	100.0
DRC	1995	2.98		Mean	2.11 - 4.36	95% CrI	Next generation matrix	Occupation	Robert 2019	100.0
**Uganda, 2000-2001**										
Uganda	20 Aug 2000 - 7 Jan 2001	1.34		Median	0.03	Standard Deviation	Compartmental model		Chowell 2004	28.6
Uganda	2000	2.70		Mean	2.5 - 4.1	95% CI			Legrand 2007	100.0
**DRC, 2012**										
DRC	2012	0.86		Mean	0.37 - 1.6	95% CrI	Branching process		Choi 2019	42.9
DRC	2012	1.11		Mean	1.06 - 1.17	95% CrI	Other		Choi 2019	42.9
DRC	2012	1.37		Mean	0.85 - 2.02	95% CrI	Branching process		Choi 2019	42.9
**West Africa 2013-2016**										
Guinea	Dec 2013 - Mar 2016	0.89		Mean			Empirical (contact tracing)	Disease generation	Robert 2019 (b)	100.0
Guinea	1 Jan 2014 - Dec 2014	1.30		Unspecified			Compartmental model		Uekermann 2019	28.6
Guinea	1 Mar 2014 - 18 Mar 2015	1.12		Mean	1.11 - 1.12	95% CI	Compartmental model		Wang 2015	42.9
Guinea	22 Mar 2014 - 29 Aug 2019	1.00		Mean	0.77 - 1.35	Range	Compartmental model		Agusto 2015	57.1
Guinea	22 Mar 2014 - 22 Mar 2015	1.26		Mean	1.22 - 1.29	95% CI	Next generation matrix		Shen 2015	71.4
Guinea	22 Mar 2014 - 29 Aug 2019	1.60		Mean	1.15 - 2.05	Range	Compartmental model		Agusto 2015	57.1
Guinea	22 Mar 2014 - 22 Aug 2014	2.46		Unspecified			Compartmental model		Fisman 2014 (b)	28.6
Guinea	22 Mar 2014 - 25 Jan 2015	4.16		Unspecified			Compartmental model		Li 2015	42.9
Guinea	25 Mar 2014 - 3 May 2015	1.21		Median	1.21 - 1.21	95% CI	Growth rate		Liu 2015	71.4
Guinea	25 Mar 2014 - 25 Oct 2014	1.49		Median	1.48 - 1.5	95% CI	Next generation matrix		Luo 2019	85.7
Guinea	27 May 2014 - 1 Jun 2015		0.85 - 1.22	Unspecified			Compartmental model	Time	Webb 2016	57.1
Guinea	Aug 2014 - May 2015	1.18		Mean	1.17 - 1.19	95% CI	Next generation matrix		Ajelli 2016	71.4
Guinea	Unspecified - 7 Sep 2014	1.24		Unspecified	1.04 - 1.42	95% CI	Other		Taylor 2016	71.4
Guinea, Liberia, Nigeria, Sierra Leone	30 Dec 2013 - 2014		1.2 - 2.02	Mean			Branching process	Region	Aylward 2014	100.0
Guinea, Liberia, Nigeria, Sierra Leone	22 Mar 2014 - 22 Aug 2014	1.78		Unspecified			Compartmental model		Fisman 2014 (b)	28.6
Guinea, Liberia, Sierra Leone	28 Dec 2013 - 3 Oct 2014	1.44	0.75 - 1.92	Other			Compartmental model		Barbarossa 2015	42.9
Guinea, Liberia, Sierra Leone	2013 - 11 Nov 2014	1.21	1.09 - 1.22	Unspecified			Branching process	Region	Evans 2015	42.9
Guinea, Liberia, Sierra Leone	2013 - 2014	1.30	0.36 - 3.37	Mean	0.64	Standard Deviation	Growth rate	Region	Krauer 2016	100.0
Guinea, Liberia, Sierra Leone	1 Jan 2014 - 28 Sep 2014	1.96		Unspecified			Other		Pruyt 2015	71.4
Guinea, Liberia, Sierra Leone	Jan 2014 - Sep 2015		0.98 - 2.39	Unspecified			Next generation matrix	Other, Region	Lin 2020	42.9
Guinea, Liberia, Sierra Leone	22 Mar 2014 - 20 Aug 2014		1.51 - 2.53	Unspecified			Compartmental model	Region	Althaus 2014	42.9
Guinea, Liberia, Sierra Leone	25 Mar 2014 - 3 May 2015	1.35		Median	1.35 - 1.35	95% CI	Growth rate		Liu 2015	71.4

Country	Survey date	Central estimate	Central range	Central type	Uncertainty	Uncertainty type	Method	Disaggregated by	Article	QA score (%)
Guinea, Liberia, Sierra Leone	Mar 2014 - Dec 2014		1.24 - 1.56	Unspecified				Region	Diaz 2018	85.7
Guinea, Liberia, Sierra Leone	6 Jul 2014 - 9 Aug 2014	1.80	1.5 - 2	Unspecified			Compartmental model		Gomes 2014	85.7
Guinea, Liberia, Sierra Leone	2014 - 2015	1.14		Unspecified			Next generation matrix		Long 2018	0.0
Guinea, Liberia, Sierra Leone	2014 - 2015	1.40		Unspecified			Next generation matrix		Denes 2019	57.1
Guinea, Liberia, Sierra Leone	2014		1.4 - 1.9	Unspecified			Compartmental model	Region	Stockdale 2021	42.9
Guinea, Liberia, Sierra Leone	Unspecified - 18 Oct 2014	1.79		Unspecified	1.78 - 1.81	95% CI	Compartmental model		Fisman 2014	28.6
Guinea, Sierra Leone	May 2014 - Jun 2014	5.92		Mean	2.97 - 11.12	HPDI 95%	Genomics		Saulnier 2017	71.4
Liberia	2013 - Dec 2014	2.00		Unspecified			Next generation matrix		Njankou 2018	57.1
Liberia	1 Jan 2014 - Apr 2015	2.50		Unspecified			Compartmental model		Uekermann 2019	28.6
Liberia	1 Mar 2014 - 18 Mar 2015	1.23		Mean	1.23 - 1.23	95% CI	Compartmental model		Wang 2015	42.9
Liberia	17 Mar 2014 - Aug 2014	2.11		Unspecified	1.88 - 2.71	Other	Next generation matrix		Valdez 2015	57.1
Liberia	18 Mar 2014 - 9 Nov 2014	1.40	0.7 - 2	Mean	0.1 - 3.9	95% CI	Compartmental model	Time	Funk 2017	100.0
Liberia	18 Mar 2014 - 9 Nov 2014	4.09		Standard Deviation	1.47 - 8.35	95% CrI	Compartmental model		Funk 2017	100.0
Liberia	23 Mar 2014 - 5 Oct 2014		0.9 - 4.6	Median			Compartmental model	Time	Enanoria 2015	85.7
Liberia	25 Mar 2014 - 3 May 2015	3.02		Median	2.61 - 3.49	95% CI	Growth rate		Liu 2015	71.4
Liberia	27 Mar 2014 - 22 Aug 2014	1.72		Unspecified			Compartmental model		Fisman 2014 (b)	28.6
Liberia	27 Mar 2014 - 25 Oct 2014	1.82		Median	1.77 - 1.83	95% CI	Next generation matrix		Luo 2019	85.7
Liberia	1 May 2014 - 1 Oct 2014	1.76	1.76 - 1.9	Unspecified			Next generation matrix		Khan 2015	28.6
Liberia	2 Jun 2014 - 2015	1.95		Unspecified			Compartmental model		Xie 2019	57.1
Liberia	5 Jun 2014 - 14 Sep 2014		1.71 - 1.94	Unspecified			Compartmental model	Other	Ponce 2019	42.9
Liberia	14 Jun 2014 - 15 Dec 2014	2.49		Unspecified	2.38 - 2.6	95% CI	Next generation matrix		Lewnard 2014	85.7
Liberia	17 Jun 2014 - 23 Sep 2014	1.54		Unspecified			Compartmental model		Webb 2015	71.4
Liberia	17 Jun 2014 - 22 Mar 2015	1.80		Mean	1.77 - 1.83	95% CI	Next generation matrix		Shen 2015	71.4
Liberia	29 Jun 2014 - 7 Oct 2014	2.01		Unspecified			Next generation matrix		Xia 2015	85.7
Liberia	Jul 2014 - Dec 2014	1.70		Median	1.1 - 2.6	95% CI	Empirical (contact tracing)		Lindblade 2015	100.0
Liberia	2014 - 2015	1.67		Median	1.29 - 2.24	95% CrI	Other		Tang 2023	42.9
Liberia	2014	2.22		Unspecified			Compartmental model		Rivers 2014	42.9
Liberia	Unspecified - 1 Oct 2014		1.54 - 1.61	Unspecified			Next generation matrix		Imran 2017	42.9
Liberia	Unspecified - 7 Sep 2014	2.06		Unspecified	1.93 - 2.27	95% CI	Other		Taylor 2016	71.4
Multi-country: Africa, Europe, USA (n = 12)	Jul 2014 - Mar 2015	0.50	0.05 - 2.9	Unspecified	0.2 - 1	90% CI	Branching process	Other	Toth 2015	71.4
Multi-country: Africa, Europe, USA (n = 8)	2014 - 2015	2.10	1.5 - 2.6	Unspecified			Compartmental model	Region	Ivorra 2015	42.9
Nigeria	17 Jul 2014 - 20 Oct 2014	3.20		Mean	0.8 - 16.4	95% CrI	Branching process		Chan 2020	85.7
Nigeria	17 Jul 2014 - 20 Oct 2014	10.00		Mean	3 - 18.5	95% CrI	Branching process		Chan 2020	85.7
Nigeria	20 Jul 2014 - 1 Oct 2014		0.4 - 12	Unspecified			Empirical (contact tracing)	Disease generation	Fasina 2014	71.4
Nigeria	20 Jul 2014 - 20 Oct 2014	9.01		Unspecified	5.22 - 15.55	95% CI	Compartmental model		Althaus 2015	71.4
Nigeria	Jul 2014 - Sep 2014	2.57		Unspecified			Next generation matrix		Do 2016	14.3
Sierra Leone	2013 - Dec 2014	2.50		Unspecified					Njankou 2018	57.1
Sierra Leone	1 Jan 2014 - Feb 2015	1.50		Unspecified			Compartmental model		Uekermann 2019	28.6
Sierra Leone	1 Mar 2014 - 18 Mar 2015	1.18		Mean	1.18 - 1.18	95% CI	Compartmental model		Wang 2015	42.9
Sierra Leone	25 Mar 2014 - 3 May 2015	1.90		Median	1.86 - 1.95	95% CI	Growth rate		Liu 2015	71.4
Sierra Leone	1 May 2014 - 1 Oct 2014	1.49	1.36 - 1.49	Unspecified			Next generation matrix		Khan 2015	28.6
Sierra Leone	12 May 2014 - 13 Nov 2015	1.30		Unspecified	1.26 - 2.53	Range	Other		Chen 2021	71.4
Sierra Leone	19 May 2014 - 11 Jan 2015	1.70		Unspecified			Compartmental model	Region	Li 2017 (b)	57.1

Country	Survey date	Central estimate	Central range	Central type	Uncertainty	Uncertainty type	Method	Disaggregated by	Article	QA score (%)
Sierra Leone	19 May 2014 - 11 Jan 2015	1.90		Unspecified			Other		Li 2020	0.0
Sierra Leone	25 May 2014 - 18 Jun 2014	2.18	1.65 - 2.18	Median	1.24 - 3.55	HPDI 95%	Genomics		Stadler 2014	100.0
Sierra Leone	25 May 2014 - 20 Jun 2014		2.1 - 3.81	Median			Compartmental model		Volz 2014	85.7
Sierra Leone	25 May 2014 - 18 Jan 2015		0.6 - 5.9	Median			Compartmental model	Time	Enanoria 2015	85.7
Sierra Leone	27 May 2014 - 1 Jun 2015		0.72 - 1.77	Unspecified			Compartmental model	Time	Webb 2016	57.1
Sierra Leone	27 May 2014 - 23 Sep 2014	1.26		Unspecified			Compartmental model		Webb 2015	71.4
Sierra Leone	27 May 2014 - 31 Aug 2014	1.29	1.29 - 1.47	Unspecified	1.27 - 1.37	95% CI	Compartmental model		Scarpino 2015	42.9
Sierra Leone	27 May 2014 - 31 Aug 2014	1.40		Unspecified	1.1 - 1.8	HPDI 95%	Genomics		Scarpino 2015	42.9
Sierra Leone	27 May 2014 - 22 Mar 2015	1.61		Mean	1.56 - 1.66	95% CI	Next generation matrix		Shen 2015	71.4
Sierra Leone	27 May 2014 - 25 Oct 2014	1.67		Median	1.59 - 1.76	95% CI	Next generation matrix		Luo 2019	85.7
Sierra Leone	27 May 2014 - 22 Aug 2014	8.33		Unspecified			Compartmental model		Fisman 2014 (b)	28.6
Sierra Leone	May 2014 - Jul 2014		1.21 - 1.6	Median				Other	Duchene 2019	57.1
Sierra Leone	May 2014 - May 2015		1.7 - 4.8	Unspecified			Compartmental model	Region	Getz 2019	71.4
Sierra Leone	Jul 2014 - Aug 2014	1.63		Mean	0 - 4	Range	Empirical (contact tracing)		Ajelli 2015	71.4
Sierra Leone	Jul 2014 - Nov 2014	2.24		Unspecified	1.52 - 4.51	95% CI	Growth rate		Ajelli 2015	71.4
Sierra Leone	Sep 2014 - 2 Feb 2015		0.5 - 8.4	Unspecified			Compartmental model	Region	Kucharski 2015	100.0
Sierra Leone	20 Oct 2014 - 30 Mar 2015	2.00		Unspecified	1.8 - 2.2	95% CI	Other		Lau 2017	85.7
Sierra Leone	20 Oct 2014 - 30 Mar 2015	2.39		Median	2.05 - 2.84	95% CrI	Other		Lau 2017 (b)	100.0
Sierra Leone	28 Nov 2014 - Feb 2015	2.90	2.1 - 4	Mean	1.2 - 6.9	90% CrI	Compartmental model	Time	Funk 2019	100.0
Sierra Leone	1 Dec 2014 - 28 Feb 2015	0.29		Mean	0.11 - 0.55		Empirical (contact tracing)	Level of exposure	Stehling-Ariza 2016	100.0
Sierra Leone	1 Dec 2014 - 28 Feb 2015	0.93		Mean	0.15 - 2.3	95% CI	Empirical (contact tracing)	Level of exposure	Stehling-Ariza 2016	100.0
Sierra Leone	2014 - 2015	1.66		Median	1.31 - 2.15	95% CrI	Other		Tang 2023	42.9
Sierra Leone	2014	1.78		Unspecified			Compartmental model		Rivers 2014	42.9
Sierra Leone	Unspecified - 7 Sep 2014	1.71		Unspecified	1.4 - 1.82	95% CI	Other		Taylor 2016	71.4
Sierra Leone	Unspecified		1.81 - 1.84	Unspecified			Next generation matrix		Imran 2017	42.9
Sierra Leone	Unspecified	2.13		Other			Next generation matrix		Abbate 2016	85.7
Unspecified	Unspecified - 6 Sep 2014		1.51 - 2.26	Unspecified			Unspecified		Hunt 2014	14.3
**DRC, 2014**										
DRC	26 Jul 2014 - 4 Oct 2014	5.15		Unspecified	3.95 - 6.69	95% CI	Compartmental model		Althaus 2015 (b)	71.4
DRC	2014	0.84		Mean	0.59 - 1.15	95% CrI			Burch 2017	42.9
**DRC, 2018-2020**										
DRC	3 May 2018 - 12 Sep 2019		1.1 - 1.21	Mean			Compartmental model	Time	Vossler 2022	42.9
DRC	5 Aug 2018 - 2 Feb 2020	1.08		Unspecified			Compartmental model		Potluri 2022	71.4
DRC	5 Aug 2018 - 20 Oct 2019	7.73		Unspecified			Next generation matrix		Mouanguissa 2021	57.1
DRC	Aug 2018 - Sep 2019		0.88 - 1.22	Median	0.76 - 1.31	95% CI	Branching process	Time	Jombart 2020	42.9
DRC	4 Aug 2019 - 10 Jan 2019	1.83		Unspecified			Compartmental model		Hart 2019	71.4
DRC	4 Aug 2019 - 10 Jan 2019	1.90		Unspecified			Compartmental model		Hart 2019	71.4
**Uganda, 2022-2023**										
Uganda	Aug 2022 - Nov 2022		1.99 - 2.7	Unspecified				Other	Marziano 2023	100.0
**Multiple outbreaks**										
DRC, Guinea, Liberia, Sierra Leone	1995 - 2016		1.53 - 266.72	Unspecified			Compartmental model	Region, Time	Siewe 2020 (b)	28.6

4.3.3 Effective reproduction number

Table S14: Effective R estimates

Country	Survey date	Central estimate	Central range	Central type	Uncertainty	Uncertainty type	Method	Disaggregated by	Article	QA score (%)
**DRC, 1995**										
DRC	1 Mar 1995 - 12 Jul 1995	0.73		Unspecified			Compartmental model		Zaman 2009	100.0
**West Africa 2013-2016**										
Guinea	22 Mar 2014 - 22 Aug 2014	1.04		Mean	0.01	Standard Deviation	Compartmental model		Kiskowski 2014	100.0
Guinea	Mar 2014 - Aug 2014	0.66		Unspecified			Branching process	Other, Region	Kucharski 2016	71.4
Guinea	Mar 2014 - Aug 2014	7.00		Unspecified			Branching process	Other, Region	Kucharski 2016	71.4
Guinea	Apr 2014 - 18 Mar 2015		1.05 - 1.31	Unspecified			Growth rate	Region, Time	Hsieh 2015	28.6
Guinea	Aug 2014 - Feb 2015	2.00		Mean			Empirical (contact tracing)		Valencia 2017	40.0
Guinea	Aug 2014 - Feb 2015	3.00		Mean			Empirical (contact tracing)		Valencia 2017	40.0
Guinea, Liberia, Sierra Leone	30 Dec 2013 - 18 Jul 2015		0 - 3.5	Unspecified			Compartmental model	Other, Region	Santermans 2016	100.0
Guinea, Liberia, Sierra Leone	Dec 2013 - 25 Nov 2014		0.93 - 1	Other			Branching process	Region, Time	Agua-Agum 2015	100.0
Guinea, Liberia, Sierra Leone	2013 - 11 Nov 2014		0.86 - 1.72	Unspecified			Branching process	Region, Time	Evans 2015	42.9
Guinea, Liberia, Sierra Leone	Jan 2014 - Feb 2015	2.30	1 - 7	Mean	1.8 - 2.7	95% CI	Unspecified	Time	Cleaton 2016	42.9
Guinea, Liberia, Sierra Leone	25 Mar 2014 - 22 Mar 2015		0.62 - 1.38	Mean			Growth rate	Region, Time	Wiratsudakul 2016	100.0
Guinea, Liberia, Sierra Leone	1 Jul 2014 - 8 Sep 2014		1.2 - 2.3	Unspecified			Compartmental model	Other, Region	Towers 2014	0.0
Guinea, Liberia, Sierra Leone	30 Dec 2014 - 31 Aug 2014		1.38 - 1.81	Mean				Region	Aylward 2014	100.0
Guinea, Liberia, Sierra Leone	2014 - 2015	1.01		Mean	0.27 - 2.58	Range	Next generation matrix		Denes 2019	57.1
Guinea, Liberia, Sierra Leone	18 Jun 2015 - 4 Aug 2015	2.58	0.64 - 4.74	Unspecified			Empirical (contact tracing)		Tiffany 2017	28.6
Liberia	22 Mar 2014 - 22 Aug 2014	1.17		Mean	0.03	Standard Deviation	Compartmental model		Kiskowski 2014	100.0
Liberia	Apr 2014 - 18 Mar 2015		1.05 - 3.58	Unspecified			Growth rate	Region, Time	Hsieh 2015	28.6
Liberia	27 May 2014 - 21 Dec 2014		0.42 - 2.9	Mean			Compartmental model	Time	Siettos 2015	14.3
Liberia	27 May 2014 - 22 Dec 2014		0.38 - 6.1	Mean			Compartmental model	Other, Time	Russo 2016	42.9
Liberia	7 Jul 2014 - 22 Sep 2014	1.73		Unspecified	1.66 - 1.83	95% CI	Branching process	Symptoms, Other	Yamin 2016	100.0
Liberia	Jul 2014 - Dec 2014	0.10		Median	0.02 - 0.6	95% CI	Empirical (contact tracing)		Lindblade 2015	100.0
Liberia	Dec 2014 - Mar 2015	0.76		Unspecified			Branching process	Other, Region	Kucharski 2016	71.4
Liberia	Dec 2014 - Mar 2015	5.00		Unspecified			Branching process	Other, Region	Kucharski 2016	71.4
Liberia, Sierra Leone	14 Apr 2014 - 11 Oct 2014		1.9 - 2.5	Unspecified			Other	Region	Majumder 2015	28.6
Sierra Leone	22 Mar 2014 - 22 Aug 2014	1.11		Mean	0.02	Standard Deviation	Compartmental model		Kiskowski 2014	100.0
Sierra Leone	27 Apr 2014 - 3 Feb 2016		0.16 - 3.31	Mean			Branching process	Time	Bodine 2018	100.0
Sierra Leone	Apr 2014 - 18 Mar 2015		1.06 - 2.12	Unspecified			Growth rate	Region, Time	Hsieh 2015	28.6
Sierra Leone	22 May 2014 - 3 Dec 2014		1.1 - 2.13	Unspecified			Compartmental model	Time	White 2015	57.1
Sierra Leone	25 May 2014 - 25 Jan 2015		0.44 - 2.22	Mean			Compartmental model	Region, Time	Yang 2015	85.7
Sierra Leone	27 May 2014 - 21 Dec 2014		1.93 - 2.5	Mean			Compartmental model	Time	Siettos 2015	14.3
Sierra Leone	May 2014 - 22 Oct 2014	0.46		Unspecified			Unspecified		Fang 2016	71.4

Country	Survey date	Central estimate	Central range	Central type	Uncertainty	Uncertainty type	Method	Disaggregated by	Article	QA score (%)
Sierra Leone	Jul 2014 - Aug 2014	2.24		Mean			Branching process		Ajelli 2015	71.4
Sierra Leone	11 Oct 2014 - Unspecified	1.30		Unspecified			Empirical (contact tracing)	Other	DeSilva 2019	40.0
Sierra Leone	11 Oct 2014 - Unspecified	4.00		Unspecified			Empirical (contact tracing)	Disease generation	DeSilva 2019	40.0
Sierra Leone	22 Oct 2014 - Sep 2015	0.09		Unspecified			Unspecified		Fang 2016	71.4
Sierra Leone	21 Dec 2014 - 15 Aug 2015		0.68 - 1.98	Mean			Compartmental model	Time	Siettos 2016	42.9
Sierra Leone	2014 - Unspecified	1.26		Unspecified	1.04 - 1.54	HPDI 95%	Genomics		Alizon 2014	0.0
Sierra Leone	18 Jan 2015		0.1 - 0.97	Median			Compartmental model	Region	Camacho 2015	100.0
Sierra Leone	Jul 2015 - Sep 2015	1.20	1.2 - 1.8	Unspecified			Empirical (contact tracing)		Glynn 2018	100.0
Sierra Leone	Oct 2015 - Jan 2016		0.83 - 9	Unspecified			Empirical (contact tracing)	Time	Kelly 2018	85.7
Sierra Leone	Sep 2016 - Jul 2017		0 - 29	Unspecified			Empirical (contact tracing)	Disease generation, Region	Kelly 2022	40.0
**DRC, 2014**										
DRC	26 Jul 2014 - 7 Oct 2014	0.84		Unspecified	-0.38 - 2.06	95% CI	Empirical (contact tracing)		Maganga 2014	28.6
DRC	26 Jul 2014 - 7 Oct 2014	1.29		Unspecified	-4.71 - 7.29	95% CI	Empirical (contact tracing)		Maganga 2014	28.6
**DRC, 2018**										
DRC	30 Apr 2018 - 24 May 2018	1.03	0.83 - 1.37	Median	0.83 - 1.37	95% CrI	Branching process		Ahuka-Mundeke 2018	100.0
**DRC, 2018-2020**										
DRC	30 Apr 2018 - 15 Jan 2019	0.90	0 - 1.8	Median	0.4 - 1.1	95% CI	Branching process		Tariq 2019	100.0
DRC	8 May 2018 - 15 Apr 2019	1.11		Mean			Branching process	Other, Region, Time	Wannier 2019	85.7
DRC	5 Aug 2018 - 27 Jul 2019	2.49		Unspecified			Next generation matrix		Juga 2020	28.6
DRC	2018 - 2020	0.33		Unspecified			Compartmental model		Ouemba Tasse 2022	0.0
DRC	Jan 2019 - Sep 2019		0.4 - 1.1	Median			Next generation matrix	Region, Time	Mizumoto 2019	100.0
DRC	Apr 2019 - Aug 2019	1.06		Mean	1.02 - 1.11	95% CI	Branching process	Other, Region, Time	Kelly 2020	85.7
DRC	12 Jun 2019 - 18 May 2020	1.14		Unspecified			Empirical (contact tracing)		Keita 2023	28.6
DRC	Jul 2019	2.19		Mean			Next generation matrix	Region, Time	Mizumoto 2019	100.0
DRC	29 Sep 2019	1.03		Median	0.59 - 2.12	95% CrI	Next generation matrix	Region	Mizumoto 2019	100.0
DRC	Sep 2019	1.20		Mean			Next generation matrix	Region, Time	Mizumoto 2019	100.0

24% of effective reproduction number estimates in Table S14 (unfiltered by QA score, n=55) were estimated using branching process models (n=13), 26% compartmental models (n=14), 11% next generation matrices (n=6), 22% contact tracing (n=12), 7% growth rate (n=4), 2% genomics (n=1) and 7% with an unspecified or "other" method (n=4).

4.3.4 Secondary attack rate

Table S15: Secondary attack rates. Note: The three Fang 2016 estimates had no units, so an assumption was made to convert them into percentages.

Country	Survey date	Central estimate (%)	Central range	Uncertainty (95% CI)	Population Sample	Sample size	Population Group	Disaggregated by	Article	QA score (%)
**DRC, 1976**										
DRC	1976		0.1 - 0.8		Unspecified		Unspecified	Unspecified	Emond 1977	0.0
**DRC, 1977**										
DRC	1 Sep 1977 - 24 Oct 1977	5.6	3.4 - 27.3		Community based		Other	Disease generation, Level of exposure, Other	International Commission 1978	40.0
**South Sudan, 1976**										
Sudan	Jun 1976 - Nov 1976	12	0.34 - 14		Household based		Persons under investigation	Disease generation	WHO/Int. Study Team 1978	60.0
Sudan	Jun 1976 - Nov 1976		23 - 81		Household based	17	Persons under investigation	Level of exposure	WHO/Int. Study Team 1978	60.0
**DRC 1981-1985***										
DRC	1981 - 1985	15	7 - 19		Contact based	188	General population	Age	Jezek 1999	20.0
**DRC, 1995**										
DRC	1 Jan 1995 - 3 Jun 1995	16			Household based	200	Persons under investigation		Dowell 1999	85.7
**Uganda, 2000-2001**										
Uganda	Aug 2000 - Jan 2001	2.5			Contact based	~1000	Persons under investigation	Age, Sex	Okware 2002	40.0
Uganda	21 Oct 2000 - 22 Dec 2000	26			Community based	73	Persons under investigation		Borchert 2011	20.0
**West Africa 2013-2016**										
Guinea	20 Sep 2014 - 31 Dec 2014		2.1 - 7.9		Community based	152	Persons under investigation	Region	Dixon 2015	80.0
Guinea, Liberia, Sierra Leone	18 Jun 2015 - 4 Aug 2015	65	33 - 89		Contact based	301	Persons under investigation	Region	Tiffany 2017	28.6
Sierra Leone	May 2014 - Sep 2015	5.9	5.6 - 6.2	5 - 7	Household based	1154	Persons under investigation	Other	Fang 2016	71.4
Sierra Leone	May 2014 - 22 Oct 2014	9.3		7.7 - 11	Household based		Persons under investigation		Fang 2016	71.4
Sierra Leone	22 Oct 2014 - Sep 2015	1.7		1 - 3.1	Household based		Persons under investigation		Fang 2016	71.4
Sierra Leone	15 Dec 2014 - 30 Apr 2015	9.9	3.3 - 26.8		Household based	838	Persons under investigation	Age, Level of exposure, Other, Sex, Symptoms, Time	Reichler 2018	80.0
Sierra Leone	Jul 2015 - Sep 2015		18 - 38		Household based	937	Household contacts of survivors	Other	Glynn 2018	100.0

4.3.5 Growth rate

Table S16: Growth rates

Country	Survey date	Central estimate	Unit	Central range	Central type	Uncertainty	Uncertainty type	Disaggregated by	Article	QA score (%)
**DRC, 1976**										
DRC	1976 - Unspecified	1.43	Per day		Other/Unspecified	0.69 - 2.62	95% CI		Viboud 2016	75.0
**West Africa 2013-2016**										
Guinea	22 Mar 2014 - 31 Aug 2014	0.01	Per day		Other/Unspecified				Weitz 2015	75.0
Guinea	25 Mar 2014 - 3 May 2015	0.02	Per day		Mean	0	Standard Deviation		Liu 2015	71.4
Guinea, Liberia, Sierra Leone	25 Mar 2014 - 3 May 2015	0.03	Per day		Mean	0	Standard Deviation		Liu 2015	71.4
Liberia	17 Mar 2014 - Aug 2014	0.05	Per day		Other/Unspecified	0	Standard Error		Valdez 2015	57.1
Liberia	25 Mar 2014 - 3 May 2015	0.09	Per day		Mean	0.01	Standard Deviation		Liu 2015	71.4
Liberia	22 Jun 2014 - 31 Aug 2014	0.05	Per day		Other/Unspecified				Weitz 2015	75.0
Sierra Leone	25 Mar 2014 - 3 May 2015	0.05	Per day		Mean	0	Standard Deviation		Liu 2015	71.4
Sierra Leone	28 May 2014 - 31 Aug 2014	0.03	Per day		Other/Unspecified				Weitz 2015	75.0
**DRC, 2018-2020**										
DRC	6 Sep 2018 - 11 Mar 2019	0.12	Per week		Median	0.11 - 0.14	95% CI		Chowell 2019	85.7
**Uganda, 2022-2023**										
Uganda	Aug 2022 - Nov 2022	0.08	Per day		Other/Unspecified	0.08 - 0.08	95% CrI		Marziano 2023	100.0
Uganda	Aug 2022 - Nov 2022	0.11	Per day		Other/Unspecified	0.05 - 0.19	95% CrI		Marziano 2023	100.0
**Multiple outbreaks**										
Uganda, Guinea, Liberia, Sierra Leone	2000 - 2014		Per week	0.08 - 2.38	Other/Unspecified			Region, Time	Viboud 2016	75.0

4.3.6 Doubling time

Table S17: Doubling times

Country	Survey date	Central estimate (Days)	Central range	Uncertainty (95% CI)	Disaggregated by	Article	QA score (%)
**West Africa 2013-2016**							
Guinea	22 Mar 2014 - 31 Aug 2014	61.0				Weitz 2015	75.0
Guinea, Liberia, Nigeria, Sierra Leone	30 Dec 2014 - 31 Aug 2014		12.84 - 59.75		Region, Time	Aylward 2014	100.0
Guinea, Liberia, Sierra Leone	Dec 2013 - 25 Nov 2014		-3322.71 - 8455.5		Region, Time	Agua-Agum 2015	100.0
Guinea, Sierra Leone	28 Sep 2014 - 11 Nov 2014	22.1		18.9 - 25.59		Tong 2015	42.9
Liberia	22 Jun 2014 - 31 Aug 2014	14.0				Weitz 2015	75.0
Sierra Leone	28 May 2014 - 31 Aug 2014	21.0				Weitz 2015	75.0

4.3.7 Overdispersion

All studies from which overdispersion estimates were extracted assumed a Negative Binomial distribution for the offspring distribution.

Table S18: Overdispersion table

Country	Survey date	Central estimate	Central range	Central type	Uncertainty	Uncertainty type	Method	Disaggregated by	Article	QA score (%)
**West Africa 2013-2016**										
Guinea	Dec 2013 - Mar 2016	0.310		Mean	0.25 - 0.37	95% CI	Empirical (contact tracing)		Robert 2019 (b)	100.0
Guinea	Mar 2014 - Aug 2014	0.190		Other/Unspecified			Branching process	Other, Region	Kucharski 2016	71.4
Guinea	Mar 2014 - Aug 2014	1.600		Other/Unspecified			Branching process	Other, Region	Kucharski 2016	71.4
Guinea, Liberia, Sierra Leone	Dec 2013 - May 2015	0.030		Other/Unspecified			Branching process		Agua-Agum 2016	100.0
Guinea, Liberia, Sierra Leone	Dec 2013 - May 2015	0.520		Other/Unspecified			Branching process		Agua-Agum 2016	100.0
Liberia	4 Jul 2014 - 2 Sep 2014	2.200		Mean	2 - 2.4	IQR	Branching process		Drake 2015	57.1
Liberia	Dec 2014 - Mar 2015	0.820		Other/Unspecified			Branching process	Other, Region	Kucharski 2016	71.4
Multi-country: Africa, Europe, USA (n = 12)	Jul 2014 - Mar 2015	0.090	0.09 - 0.5	Other/Unspecified	0.03 - 0.2	90% CI	Branching process	Other	Toth 2015	71.4
Sierra Leone	Jul 2014 - Aug 2014	0.450		Other/Unspecified	0.19 - 1.32	95% CI	Empirical (contact tracing)		Ajelli 2015	71.4
Sierra Leone	4 Aug 2014 - 29 Mar 2015	0.065		Mean	0.037 - 0.11	HPDI 95%	Branching process	Time	Zhang 2022	57.1
Sierra Leone	20 Oct 2014 - 30 Mar 2015	0.370		Mean			Other		Lau 2017 (b)	100.0
Sierra Leone	20 Oct 2014 - 30 Mar 2015	0.470		Other/Unspecified			Other		Lau 2017	85.7
**DRC, 2018-2020**										
DRC	31 Jul 2018 - 26 Apr 2020	0.270		Other/Unspecified	0.2 - 0.33	95% CrI	Empirical (contact tracing)		Polonsky 2021	100.0
**Multiple outbreaks**										
Multi-country: Africa (n = 6)	2000 - 2015	0.240	0.24 - 2.09	Median	0.06	Standard Error	Empirical (contact tracing)	Other	Taube 2022	71.4

4.3.8 Risk factors associated with transmission

Table S19: Risk factors for infection table. Total is the number of parameters extracted for each risk factor for infection.

Risk Factor for Infection	Significant			Not significant			Unspecified significance			Total
	Adjusted	Not adjusted	Unspecified	Adjusted	Not adjusted	Unspecified	Adjusted	Not adjusted	Unspecified	
Close contact	6	4	5	2	1	2	0	0	2	22
Age	5	2	2	0	4	3	1	0	0	17
Sex	2	1	0	5	4	3	1	0	0	16
Funeral	2	3	2	1	2	1	0	0	3	14
Household contact	3	2	0	0	0	1	0	0	1	7
Occupation	2	2	0	1	1	1	0	0	0	7
Non-household contact	1	1	0	0	0	0	0	0	1	3
Hospitalisation	0	2	0	0	0	0	0	0	1	3
Contact with animal	0	0	0	0	1	1	0	0	0	2
Social gathering	0	0	0	0	1	0	0	0	0	1
Other	8	5	5	6	3	6	1	1	2	37

Table S20: Risk factors for onward transmission by significance and adjustment.

Risk Factor for Onward Transmission	Significant	Not significant
	Adjusted	
Age	1	0
First generation of transmission chain	1	0
Funeral	1	0
Hospitalisation	1	0
Sex	1	0
Socioeconomic status	1	0
Survival	1	0
Location (rural/urban)	0	1

4.4 Temporal dynamics of disease progression

4.4.1 Time periods for the infection process

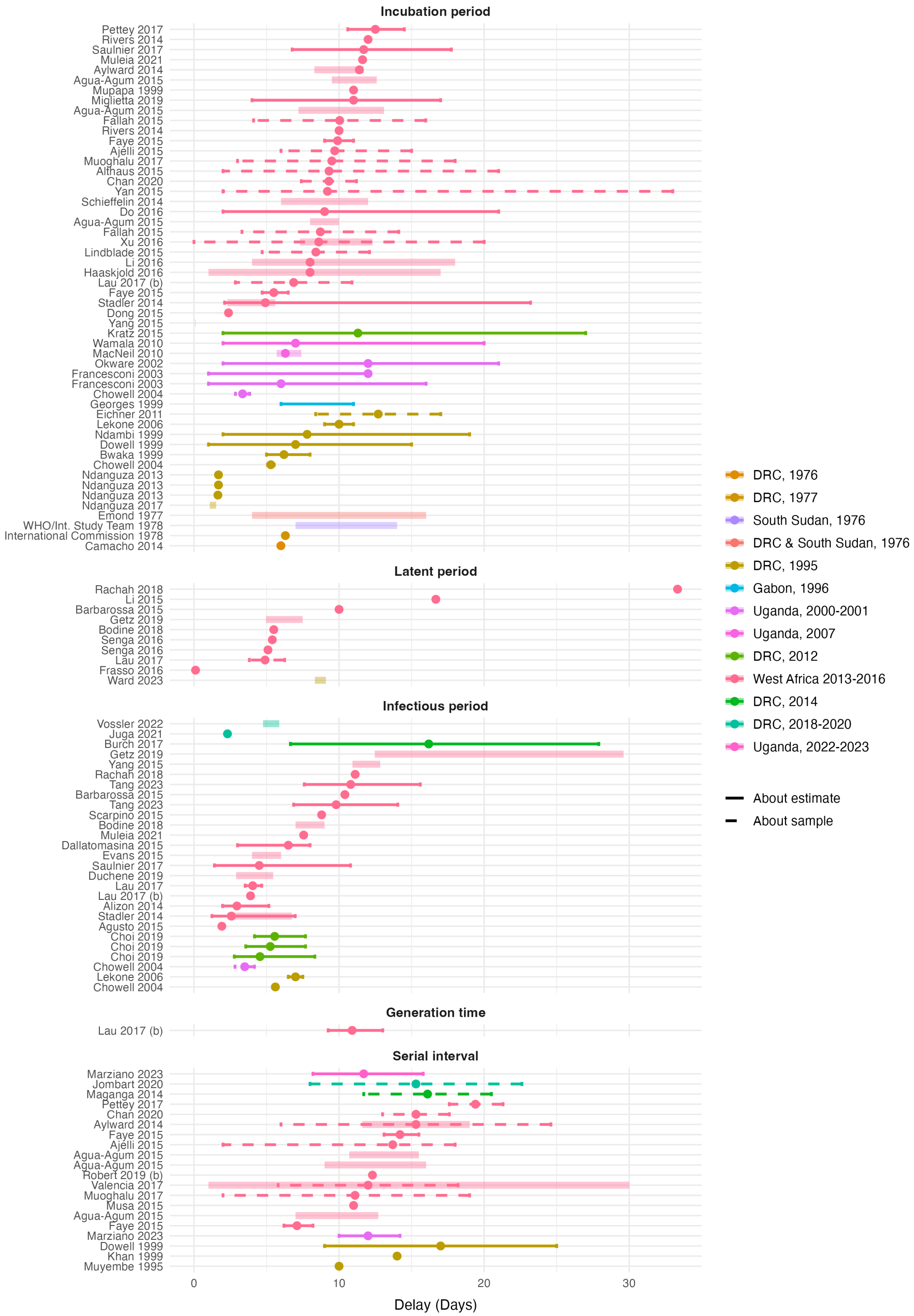

Figure S2: Time periods for the infection process, including the incubation period, latent period, infectious period, generation time and serial interval. This figure excludes one outlier (Martinez et al, 2022). Error bars show uncertainty around the estimate (solid lines) and sample (dashed lines).

4.4.2 Delays beyond symptom onset

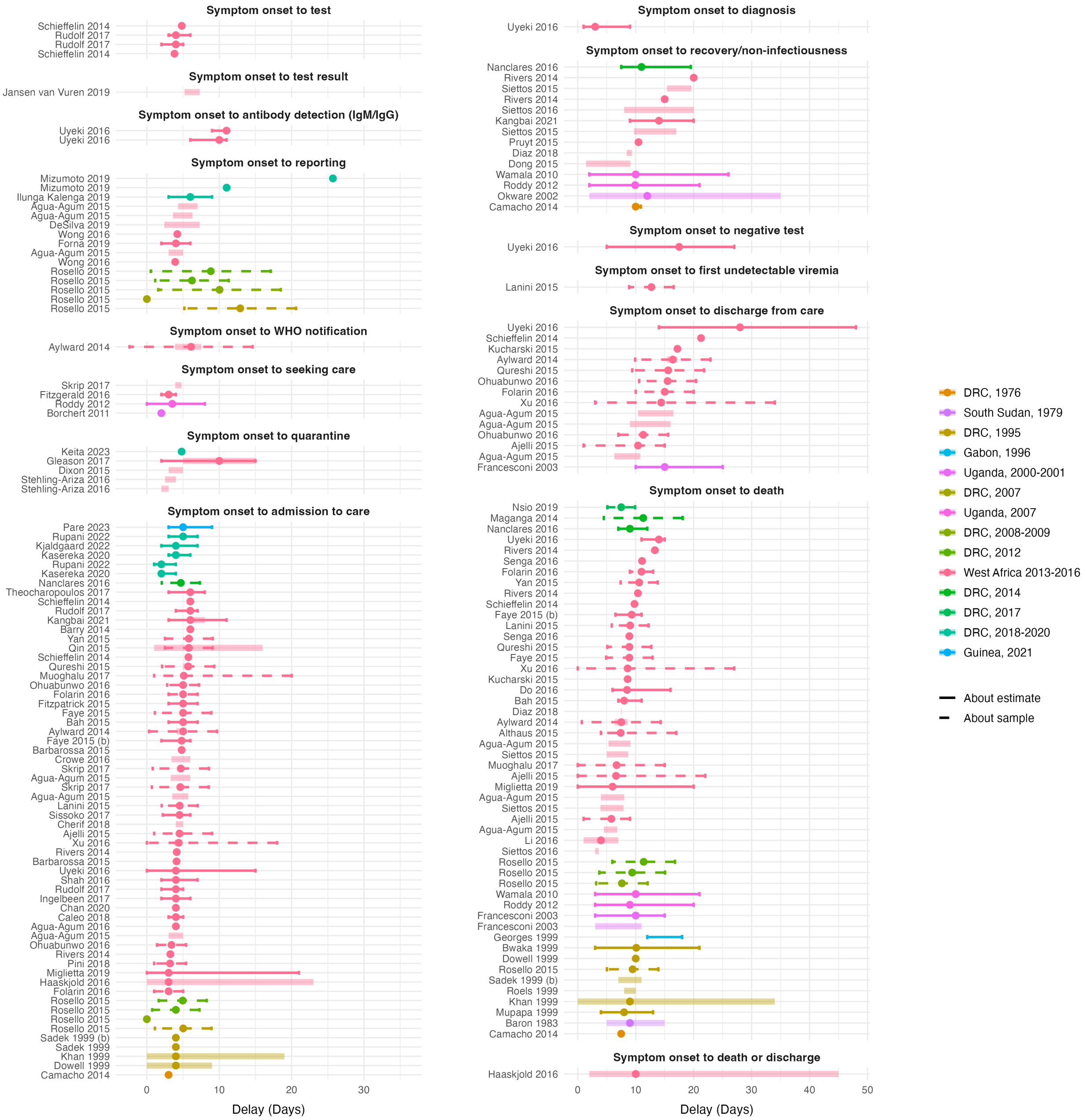

Figure S3: All delays from symptom onset to various endpoints, including test, test result, antibody detection, reporting, WHO notification, seeking care, quarantine, admission to care, diagnosis, recovery/non-infectiousness, negative test, undetectable viremia, discharge from care, death, and discharge or death (when no distinction was made between these outcomes). Error bars show uncertainty around the estimate (solid lines) and sample (dashed lines).

4.4.3 Delays beyond admission to care

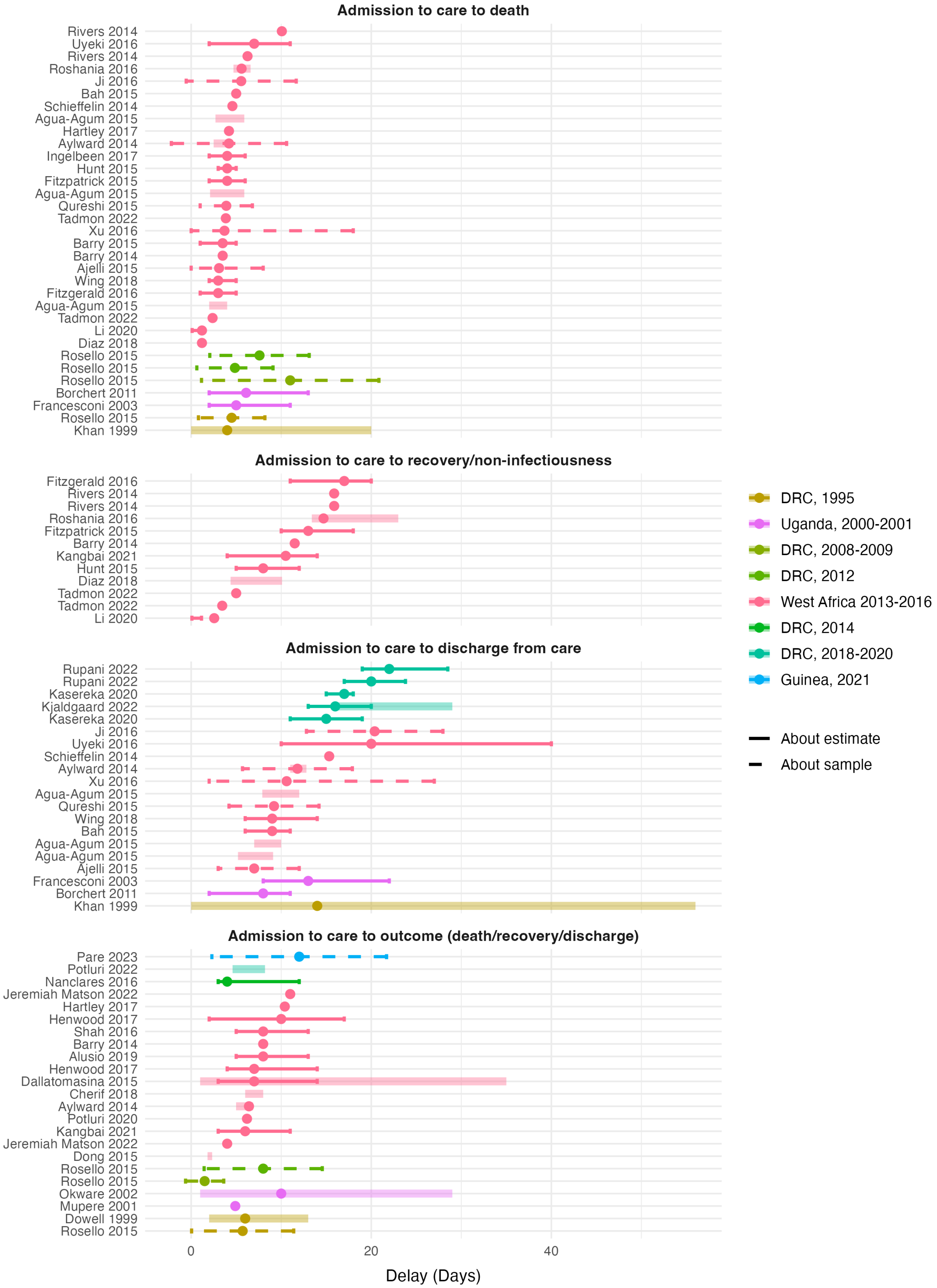

Figure S4: Delays beyond admission to care. Error bars show uncertainty around the estimate (solid lines) and sample (dashed lines)

4.4.4 QA unfiltered meta-analysis by Ebola virus species

A) Incubation period

Study or Subgroup	Mean	SD	Total	Weight (common)	Weight (random)	Mean IV, Fixed + Random, 95% CI
**Ebola virus species = Zaire**						
Ajelli 2015	9.70	3.70	8	0.01%	7.15%	9.70 [7.14; 12.26]
Althaus 2015	9.31	5.28	20	0.01%	7.59%	9.31 [6.99; 11.63]
Chan 2020	9.30	1.90	20	0.07%	9.94%	9.30 [8.47; 10.13]
Chowell 2004	5.30	0.23	315	76.82%	10.42%	5.30 [5.27; 5.33]
Dowell 1999	7.28	3.50	27	0.03%	9.29%	7.28 [5.96; 8.61]
Muoghalu 2017	9.50	4.00	76	0.06%	9.86%	9.50 [8.60; 10.40]
Xu 2016	8.60	6.10	31	0.01%	7.89%	8.60 [6.45; 10.75]
Yan 2015	9.20	6.70	49	0.01%	8.37%	9.20 [7.32; 11.08]
Total (common effect, 95% CI)			546	77.02%	.	5.31 [5.28; 5.34]
Total (random effect, 95% CI)			.		70.52%	8.36 [7.21; 9.52]
Heterogeneity: $Tau^2 = 2.1488$; $Chi^2 = 229.02$, df = 7 ($P < 0.01$); $I^2 = 97\%$						
**Ebola virus species = Sudan**						
Chowell 2004	3.35	0.49	425	22.84%	10.41%	3.35 [3.30; 3.40]
Francesconi 2003	6.58	3.85	24	0.02%	8.94%	6.58 [5.04; 8.12]
Total (common effect, 95% CI)			449	22.86%	.	3.35 [3.31; 3.40]
Total (random effect, 95% CI)			.		19.36%	4.77 [2.57; 6.98]
Heterogeneity: $Tau^2 = 2.2591$; $Chi^2 = 16.85$, df = 1 ($P < 0.01$); $I^2 = 94\%$						
**Ebola virus species = Bundibugyo**						
Wamala 2010	7.68	3.53	116	0.12%	10.13%	7.68 [7.04; 8.32]
**Total (common effect, 95% CI)**			**1111**	**100.00%**	**.**	**4.87 [4.84; 4.89]**
**Total (random effect, 95% CI)**			**.**		**100.00%**	**7.65 [6.43; 8.88]**
Heterogeneity: $Tau^2 = 3.7533$; $Chi^2 = 5552.03$, df = 10 ($P = 0$); $I^2 = 100\%$						
Test for subgroup differences (common effect): $Chi^2 = 5306.15$, df = 2 ($P = 0$)						
Test for subgroup differences (random effects): $Chi^2 = 8.02$, df = 2 ($P = 0.02$)						

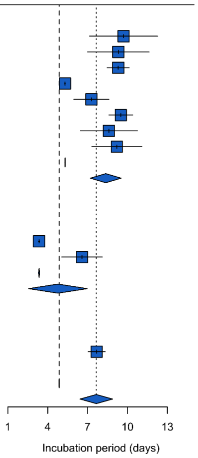

B) Time from symptom onset to death

Study or Subgroup	Mean	SD	Total	Weight (common)	Weight (random)	Mean IV, Fixed + Random, 95% CI
**Ebola virus species = Zaire**						
Althaus 2015	7.41	3.24	20	1.68%	4.70%	7.41 [5.99; 8.83]
Aylward 2014	7.50	6.80	594	11.29%	5.62%	7.50 [6.95; 8.05]
Bah 2015	8.72	3.23	16	1.35%	4.50%	8.72 [7.14; 10.30]
Faye 2015	8.90	4.00	82	4.51%	5.34%	8.90 [8.03; 9.77]
Folarin 2016	11.00	2.00	8	1.76%	4.75%	11.00 [9.61; 12.39]
Lanini 2015	9.04	3.17	84	7.35%	5.52%	9.04 [8.36; 9.72]
Maganga 2014	11.30	6.80	32	0.61%	3.52%	11.30 [8.94; 13.66]
Miglietta 2019	6.44	3.34	454	35.79%	5.75%	6.44 [6.13; 6.74]
Muoghalu 2017	6.70	4.10	27	1.41%	4.54%	6.70 [5.15; 8.25]
Nanclares 2016	9.79	3.92	25	1.43%	4.55%	9.79 [8.25; 11.32]
Qureshi 2015	8.90	3.80	70	4.26%	5.32%	8.90 [8.01; 9.79]
Rosello 2015	9.47	4.44	317	14.14%	5.65%	9.47 [8.98; 9.96]
Rosello 2015	7.62	4.44	32	1.43%	4.55%	7.62 [6.08; 9.16]
Rosello 2015	9.39	5.67	68	1.86%	4.79%	9.39 [8.04; 10.74]
Schieffelin 2014	9.79	4.32	38	1.79%	4.76%	9.79 [8.42; 11.16]
Uyeki 2016	13.44	1.72	5	1.49%	4.59%	13.44 [11.93; 14.94]
Xu 2016	8.60	4.80	76	2.90%	5.12%	8.60 [7.52; 9.68]
Total (common effect, 95% CI)			1948	95.04%	.	7.98 [7.79; 8.17]
Total (random effect, 95% CI)			.		83.57%	8.98 [8.20; 9.76]
Heterogeneity: $Tau^2 = 2.2886$; $Chi^2 = 251.41$, df = 16 ($P < 0.01$); $I^2 = 94\%$						
**Ebola virus species = Sudan**						
Francesconi 2003	9.54	3.46	15	1.10%	4.28%	9.54 [7.79; 11.29]
**Ebola virus species = Bundibugyo**						
Roddy 2012	9.90	5.36	11	0.34%	2.67%	9.90 [6.73; 13.06]
Rosello 2015	11.37	5.41	52	1.56%	4.64%	11.37 [9.90; 12.84]
Wamala 2010	10.58	4.19	39	1.96%	4.84%	10.58 [9.27; 11.90]
Total (common effect, 95% CI)			102	3.86%	.	10.84 [9.91; 11.78]
Total (random effect, 95% CI)			.		12.15%	10.84 [9.91; 11.78]
Heterogeneity: $Tau^2 = 0$; $Chi^2 = 0.99$, df = 2 ($P = 0.61$); $I^2 = 0\%$						
**Total (common effect, 95% CI)**			**2065**	**100.00%**	**.**	**8.10 [7.92; 8.29]**
**Total (random effect, 95% CI)**			**.**		**100.00%**	**9.22 [8.51; 9.92]**
Heterogeneity: $Tau^2 = 2.2221$; $Chi^2 = 289.62$, df = 20 ($P < 0.01$); $I^2 = 93\%$						
Test for subgroup differences (common effect): $Chi^2 = 37.22$, df = 2 ($P < 0.01$)						
Test for subgroup differences (random effects): $Chi^2 = 9.00$, df = 2 ($P = 0.01$)						

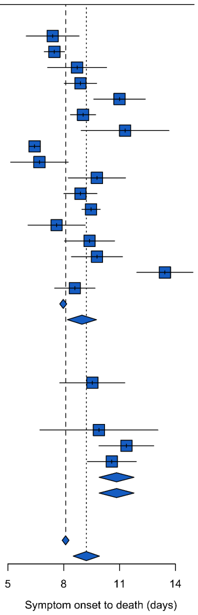

C) Time from symptom onset to recovery

Study or Subgroup	Mean	SD	Total	Weight (common)	Weight (random)	Mean IV, Fixed + Random, 95% CI
**Ebola virus species = Zaire**						
Kangbai 2021	16.29	8.26	105	28.40%	28.11%	16.29 [14.71; 17.87]
Nanclares 2016	14.82	9.42	25	5.21%	19.14%	14.82 [11.13; 18.51]
Total (common effect, 95% CI)			130	33.61%	.	16.06 [14.61; 17.52]
Total (random effect, 95% CI)			.		47.24%	16.06 [14.61; 17.52]
Heterogeneity: $Tau^2 = 0$; $Chi^2 = 0.51$, df = 1 ($P = 0.47$); $I^2 = 0\%$						
**Ebola virus species = Bundibugyo**						
Roddy 2012	10.47	5.47	15	9.25%	23.08%	10.47 [7.70; 13.24]
Wamala 2010	10.82	4.99	77	57.14%	29.67%	10.82 [9.70; 11.93]
Total (common effect, 95% CI)			92	66.39%	.	10.77 [9.74; 11.80]
Total (random effect, 95% CI)			.		52.76%	10.77 [9.74; 11.80]
Heterogeneity: $Tau^2 = 0$; $Chi^2 = 0.05$, df = 1 ($P = 0.82$); $I^2 = 0\%$						
**Total (common effect, 95% CI)**			**222**	**100.00%**	**.**	**12.55 [11.71; 13.39]**
**Total (random effect, 95% CI)**			**.**		**100.00%**	**13.04 [10.46; 15.63]**
Heterogeneity: $Tau^2 = 5.5347$; $Chi^2 = 34.42$, df = 3 ($P < 0.01$); $I^2 = 91\%$						
Test for subgroup differences (common effect): $Chi^2 = 33.86$, df = 1 ($P < 0.01$)						
Test for subgroup differences (random effects): $Chi^2 = 33.86$, df = 1 ($P < 0.01$)						

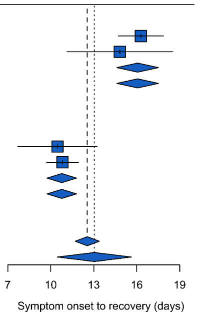

Figure S5: Meta-analysis for A) incubation period B) onset-to-death and C) onset-to-recovery regardless of QA score, with a species sub-group analysis

4.4.5 QA unfiltered meta-analysis

A) Infectious period

Study	Mean	SD	Total	Weight (common)	Weight (random)	Mean IV, Fixed + Random, 95% CI
Chowell 2004	5.61	0.19	315	89.88%	33.60%	5.61 [5.59; 5.63]
Chowell 2004	3.50	0.67	425	9.75%	33.57%	3.50 [3.44; 3.56]
Dallatomasina 2015	6.02	3.72	489	0.36%	32.83%	6.02 [5.69; 6.35]
**Total (common effect, 95% CI)**			**1229**	**100.00%**	.	**5.41 [5.39; 5.43]**
**Total (random effect, 95% CI)**				.	**100.00%**	**5.04 [3.79; 6.29]**

Heterogeneity: $Tau^2 = 1.2111$; $Chi^2 = 3815.78$, df = 2 (P = 0); $I^2 = 100\%$

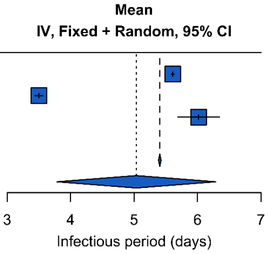

B) Serial interval

Study	Mean	SD	Total	Weight (common)	Weight (random)	Mean IV, Fixed + Random, 95% CI
Ajelli 2015	13.70	4.50	12	0.95%	9.26%	13.70 [11.15; 16.25]
Aylward 2014	15.30	9.30	92	1.70%	10.42%	15.30 [13.40; 17.20]
Chan 2020	15.30	2.30	20	6.03%	11.76%	15.30 [14.29; 16.31]
Dowell 1999	17.14	2.93	200	37.30%	12.27%	17.14 [16.74; 17.55]
Jombart 2020	15.30	7.30	1426	42.70%	12.29%	15.30 [14.92; 15.68]
Maganga 2014	16.10	4.40	28	2.31%	10.88%	16.10 [14.47; 17.73]
Muoghalu 2017	11.10	5.20	47	2.77%	11.10%	11.10 [9.61; 12.59]
Pettey 2017	19.40	5.10	28	1.72%	10.44%	19.40 [17.51; 21.29]
Valencia 2017	12.00	6.20	109	4.53%	11.57%	12.00 [10.84; 13.16]
**Total (common effect, 95% CI)**			**1962**	**100.00%**	.	**15.79 [15.55; 16.04]**
**Total (random effect, 95% CI)**				.	**100.00%**	**15.05 [13.50; 16.59]**

Heterogeneity: $Tau^2 = 5.0110$; $Chi^2 = 146.03$, df = 8 (P < 0.01); $I^2 = 95\%$

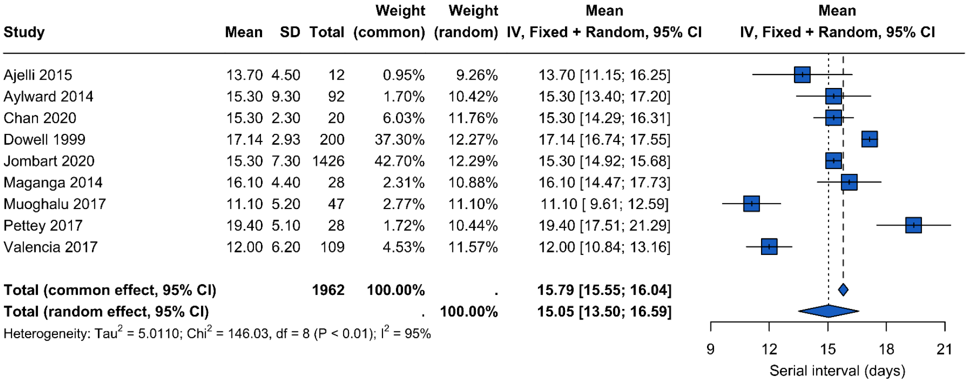

Figure S6: Meta-analysis for A) infectious period and B) serial interval regardless of QA score

4.5 Severity

4.5.1 Case Fatality Ratio (CFR)

Table S21: Case fatality ratio results

Country	Survey date	Central estimate	Deaths	Cases	Central range	Uncertainty	Uncertainty type	Adjustment	Population Sample	Disaggregated by	Article	QA score (%)
**DRC, 1976**												
DRC	Aug 1976 - Nov 1976	88				80 - 94	95% CI	Unspecified	Population based		Camacho 2014	71.4
DRC	Aug 1976 - Oct 1976	96	252	262		93 - 98	95% CI	Adjusted	Unspecified	Age, Time	Rosello 2015	100.0
DRC	1 Sep 1976 - 24 Oct 1976	88	280	318				Unspecified	Hospital based		International Commission 1978	40.0
**South Sudan, 1976**												
Sudan	Jun 1976 - Nov 1976	53	151	284	35 - 67			Naive	Hospital based	Age, Region, Sex, Time	WHO/Int. Study Team 1978	60.0
**South Sudan, 1979**												
South Sudan	31 Jul 1979 - 6 Oct 1979	65	22	34	0 - 100			Unspecified	Hospital based	Time	Baron 1983	14.3
**DRC 1981-1985***												
DRC	1981 - 1985	43	9	21				Naive	Hospital based		Jezek 1999	20.0
**Gabon, 1994**												
Gabon	Dec 1994 - Feb 1995	59	29	49				Unspecified	Hospital based		Georges 1999	80.0
**DRC, 1995**												
DRC	1 Jan 1995 - 3 Jun 1995	95	52	55				Unspecified	Household based		Dowell 1999	85.7
DRC	6 Jan 1995 - 16 Jul 1995	81	250	310				Unspecified	Community based		Khan 1999	42.9
DRC	13 Jan 1995 - 24 Aug 1995	77						Unspecified	Population based		Butler 1996	40.0
DRC	Jan 1995 - Jun 1995	78	248	317		73 - 83	95% CI	Adjusted	Unspecified	Age, Time	Rosello 2015	100.0
DRC	1 Mar 1995 - 16 Jul 1995				82 - 83			Unspecified	Population based	Other	Ndanguza 2017	57.1
DRC	9 Apr 1995 - 23 May 1995	74	101	136				Naive	Population based		Muyembe 1995	50.0
DRC	Apr 1995 - Jun 1995	96	14	15				Adjusted	Hospital based		Mupapa 1999	20.0
DRC	Apr 1995 - Unspecified	78	18	23				Unspecified	Hospital based		Ndambi 1999	100.0
DRC	May 1995	81	250	310	69 - 96			Naive	Hospital based	Age, Occupation, Other, Sex, Time	Sadek 1999 (b)	60.0
DRC	1995				54 - 83			Naive	Unspecified	Other	Roels 1999	40.0
**Gabon, 1996**												
Gabon	Feb 1996 - Unspecified		21	37				Naive	Population based		Amblard 1997	20.0
Gabon	Feb 1996 - Unspecified	68	21	31				Unspecified	Population based		Georges 1999	80.0
Gabon	Jul 1996 - Dec 1996		40	52				Naive	Population based		Amblard 1997	20.0
Gabon	Oct 1996 - Mar 1997	75	45	60				Unspecified	Population based		Georges 1999	80.0
**Uganda, 2000-2001**												
Uganda	Aug 2000 - Jan 2001	53	224	425				Unspecified			Okware 2002	40.0
Uganda	8 Oct 2000 - 27 Feb 2001	53	224	425	52 - 80			Naive	Population based	Region	Lamunu 2004	40.0
Uganda	21 Oct 2000 - 22 Dec 2000	69	18	26				Naive	Community based	Other	Borchert 2011	20.0
Uganda	Oct 2000 - Feb 2001	40	8	20				Adjusted	Hospital based	Other	Mupere 2001	40.0
Uganda	Oct 2000 - Feb 2001	28	18	64				Adjusted	Hospital based	Other	Mupere 2001	40.0
Uganda	Oct 2000 - Feb 2001	8	7	84				Adjusted	Hospital based	Other	Mupere 2001	40.0
Uganda	2000 - 2001				29 - 77			Unspecified	Unspecified	Age	McElroy 2014	100.0
Uganda	2000				0 - 100			Unspecified			Okware 2015	20.0
**Republic of the Congo, 2005**												
Republic of the Congo	18 Apr 2005 - 8 Jul 2005	83	10	12				Unspecified	Community based	Region	Nkoghe 2011 (b)	42.9
**DRC, 2007**												
DRC	Apr 2007 - Oct 2007	74	187			68 - 79	95% CI	Adjusted	Unspecified	Age, Time	Rosello 2015	100.0
**Uganda, 2007**												
Uganda	Aug 2007 - Dec 2007	32						Unspecified			MacNeil 2011	42.9
Uganda	29 Nov 2007 - 20 Feb 2008	42	11	26				Naive	Hospital based		Roddy 2012	57.1
Uganda	29 Nov 2007 - 20 Feb 2008	25	37	149				Naive	Population based		Roddy 2012	57.1
Uganda	29 Nov 2007 - 20 Feb 2008	34	39	116	29 - 100			Unspecified	Community based	Region, Sex, Other	Wamala 2010	80.0
Uganda	2007 - Unspecified	40	17	43				Adjusted	Community based		MacNeil 2010	85.7
**DRC, 2008-2009**												
DRC	Nov 2008 - Jan 2009	44	14	32		26 - 62	95% CI	Adjusted	Unspecified	Age, Time	Rosello 2015	100.0

Country	Survey date	Central estimate	Deaths	Cases	Central range	Uncertainty	Uncertainty type	Adjustment	Population Sample	Disaggregated by	Article	QA score (%)
**DRC, 2012**												
DRC	Jun 2012 - Nov 2012	54	28	52		39 - 68	95% CI	Adjusted	Unspecified	Age, Time	Rosello 2015	100.0
DRC	2 Aug 2012 - 26 Nov 2012	54	28	52	0 - 100			Adjusted	Other	Age, Other, Sex	Kratz 2015	71.4
DRC	Aug 2012 - Oct 2012	74	49	66		62 - 84	95% CI	Adjusted	Unspecified	Age, Time	Rosello 2015	100.0
**West Africa 2013-2016**												
Guinea	Dec 2013 - 25 Nov 2014	66						Unspecified	Community based		Helleringer 2015	100.0
Guinea	1 Jan 2014 - 29 Mar 2015	46	429	932	40 - 60	43 - 49	95% CI	Adjusted	Hospital based	Region	Rico 2016	80.0
Guinea	1 Jan 2014 - 29 Mar 2015	60	817	1,355	53 - 76			Naive	Community based	Region	Rico 2016	80.0
Guinea	1 Jan 2014 - 29 Mar 2015	40	168	420	23 - 44	35 - 45	95% CI	Adjusted	Hospital based	Region	Rico 2016	80.0
Guinea	1 Jan 2014 - 29 Mar 2015	53	295	553	44 - 60			Naive	Community based	Region	Rico 2016	80.0
Guinea	Jan 2014 - Dec 2015	63	437	695				Adjusted	Hospital based		Cherif 2017	100.0
Guinea	Jan 2014 - Dec 2015	68	1,574	2,313	66 - 81			Adjusted	Hospital based	Age	Cherif 2018	100.0
Guinea	10 Feb 2014 - 11 Aug 2014	54				49 - 63	95% CI	Unspecified	Community based		Faye 2015	85.7
Guinea	1 Mar 2014 - 28 Feb 2015				35 - 49			Unspecified	Hospital based	Time	Faye 2015 (b)	71.4
Guinea	17 Mar 2014 - 29 Mar 2015	60	719	1,205				Naive	Hospital based	Age, Other	Kerber 2016	40.0
Guinea	22 Mar 2014 - 25 Jan 2015	66						Unspecified	Unspecified		Li 2015	42.9
Guinea	25 Mar 2014 - 26 Apr 2014	43	16	37				Adjusted	Hospital based		Bah 2015	28.6
Guinea	25 Mar 2014 - 20 Aug 2014	44	39	90		33 - 54	95% CI	Naive	Hospital based		Barry 2014	14.3
Guinea	Mar 2014 - Unspecified	71						Unspecified	Hospital based		Baize 2014	42.9
Guinea	Mar 2014 - Unspecified	86						Unspecified	Hospital based		Baize 2014	42.9
Guinea	Mar 2014 - Aug 2014	44	39	89				Naive	Hospital based		Barry 2015	57.1
Guinea	Mar 2014 - May 2015	48	70	146	23 - 74			Unspecified	Hospital based	Other, Region, Sex, Symptoms	Sow 2020	80.0
Guinea	20 Sep 2014 - 31 Dec 2014	81	123	152	79 - 84			Naive	Community based	Region	Dixon 2015	80.0
Guinea	29 Nov 2014 - 31 Jan 2015	59						Unspecified	Hospital based		Vernet 2017	71.4
Guinea	1 Dec 2014 - 1 Mar 2015				19 - 81			Unspecified	Population based	Other	Dong 2015	14.3
Guinea	2 Dec 2014 - 23 Feb 2015	61	46	76				Naive	Hospital based		Loubet 2016	0.0
Guinea	22 Jun 2017 - 9 Jul 2017	56	10	18		31 - 78	95% CI	Unspecified	Household based		Timothy 2019	100.0
Guinea, Liberia, Nigeria, Sierra Leone	30 Dec 2013 - 14 Sep 2014	71			32 - 84	69 - 73	95% CI	Adjusted		Age, Occupation, Region, Sex, Symptoms, Time, Other	Aylward 2014	100.0
Guinea, Liberia, Sierra Leone	28 Dec 2013 - 3 Oct 2014	73						Unspecified	Community based		Barbarossa 2015	42.9
Guinea, Liberia, Sierra Leone	28 Dec 2013 - 3 Oct 2014	61						Unspecified	Hospital based		Barbarossa 2015	42.9
Guinea, Liberia, Sierra Leone	30 Dec 2013 - 28 Sep 2015	63			40 - 86	62 - 64	95% CI	Adjusted		Age, Other, Region, Time	Garske 2016	100.0
Guinea, Liberia, Sierra Leone	30 Dec 2013 - 8 Jul 2015				19 - 66			Unspecified	Population based	Region	Santermans 2016	100.0
Guinea, Liberia, Sierra Leone	Dec 2013 - 25 Nov 2014				24 - 58			Naive	Population based	Region, Time	Agua-Agum 2015	100.0
Guinea, Liberia, Sierra Leone	Dec 2013 - 25 Nov 2014				52 - 100			Adjusted	Population based	Age, Occupation, Other, Region, Sex, Time	Agua-Agum 2015	100.0
Guinea, Liberia, Sierra Leone	2013 - 2016	83		33,338		46 - 86	95% CI	Adjusted	Population based	Age, Other, Region, Symptoms	Forna 2019	100.0
Guinea, Liberia, Sierra Leone	2013 - 2016	72		33,338		56 - 80	95% CI	Naive	Population based	Age, Other, Region, Symptoms	Forna 2019	100.0
Guinea, Liberia, Sierra Leone	2013 - 2016	75		18,644		74 - 77	95% CI	Naive	Population based	Age, Other, Region, Symptoms	Forna 2019	100.0
Guinea, Liberia, Sierra Leone	Jan 2014 - Feb 2015	74				68 - 80	95% CI	Unspecified		Age, Level of exposure, Region, Time	Cleaton 2016	42.9
Guinea, Liberia, Sierra Leone	22 Mar 2014 - 20 Aug 2014				48 - 74			Unspecified	Unspecified	Region	Althaus 2014	42.9
Guinea, Liberia, Sierra Leone	24 May 2014 - 30 Sep 2014				39 - 67			Unspecified	Population based	Region	D'Silva 2017	71.4
Liberia	Dec 2013 - 25 Nov 2014	43						Unspecified	Community based		Helleringer 2015	100.0
Liberia	28 Feb 2014 - 1 Dec 2014				42 - 47			Unspecified			Fallah 2015	42.9
Liberia	20 Mar 2014 - 20 Sep 2014	53	330	619				Naive			Ki 2015	0.0
Liberia	20 Mar 2014 - 20 Sep 2014	82						Unspecified			Ki 2015	0.0
Liberia	21 Mar 2014 - 31 Dec 2014	54	229	428		49 - 58	95% CI	Naive		Other	Weppelmann 2016	100.0
Liberia	4 Apr 2014 - 29 Mar 2015	69	2,701	3,897		68 - 71	95% CI	Naive	Population based	Age, Region, Sex, Time	Furuse 2017	100.0
Liberia	14 May 2014 - 7 Mar 2015	59	10	17				Unspecified	Community based		Kuehne 2016	28.6
Liberia	27 May 2014 - 22 Dec 2014				34 - 83			Unspecified	Unspecified	Other, Time	Russo 2016	42.9
Liberia	27 May 2014 - 21 Dec 2014				12 - 70			Adjusted	Population based	Time	Siettos 2015	14.3
Liberia	7 Jul 2014 - 22 Sep 2014	55	76	139	9 - 93	60 - 64	95% CI	Naive		Age, Other, Sex, Symptoms	Yamin 2016	100.0

Country	Survey date	Central estimate	Deaths	Cases	Central range	Uncertainty	Uncertainty type	Adjustment	Population Sample	Disaggregated by	Article	QA score (%)
Liberia	Jul 2014 - Dec 2014	68			50 - 92	60 - 74	95% CI	Unspecified	Hospital based	Age, Other, Time	Lindblade 2015	100.0
Liberia	28 Aug 2014 - 18 Dec 2014				33 - 80			Unspecified	Hospital based	Other	Jeremiah Matson 2022	100.0
Liberia	15 Sep 2014 - 4 Jan 2015	51	82	160				Naive	Hospital based		Levine 2015	100.0
Liberia	2014	50						Unspecified	Unspecified		Rivers 2014	42.9
Liberia, Sierra Leone	15 Sep 2014 - 31 Dec 2015	58	244	424				Unspecified	Hospital based		Alusio 2019	100.0
Liberia, Sierra Leone	15 Sep 2014 - 15 Sep 2015	46	6	13				Adjusted	Hospital based	Other	Henwood 2017	71.4
Liberia, Sierra Leone	15 Sep 2014 - 15 Sep 2015	54	87	162				Unspecified	Hospital based	Other	Henwood 2017	71.4
Liberia, Sierra Leone	15 Sep 2014 - 15 Sep 2015	58	268	465	38 - 93			Adjusted	Hospital based	Age, Region, Sex	Roshania 2016	100.0
Liberia, Sierra Leone	Sep 2014 - Sep 2015	58	244	424				Unspecified	Hospital based	Other	Garbern 2019	50.0
Liberia, Sierra Leone	Sep 2014 - Sep 2015	58						Unspecified	Hospital based	Age, Other, Region, Sex	Peters 2019	85.7
Multi-country: Africa, Europe, USA (n = 10)	Dec 2013 - Mar 2016	40	11,325	28,652				Unspecified		Occupation, Region	Shultz 2016	42.9
Multi-country: Africa, Europe, USA (n = 6)	Jan 2014 - 12 Oct 2014	55	425	236				Unspecified	Unspecified		Hossain 2016	0.0
Multi-country: Europe & USA (n = 9)	Aug 2014 - Dec 2015	18	5	27				Naive	Hospital based		Uyeki 2016	80.0
Nigeria	20 Jul 2014 - 20 Oct 2014	39				14 - 71	95% CrI	Unspecified	Unspecified		Althaus 2015	71.4
Nigeria	20 Jul 2014 - 1 Oct 2014	40	8	20		22 - 61	95% CI	Unspecified	Population based		Fasina 2014	71.4
Nigeria	Jul 2014 - Unspecified	40	7	20	22 - 46			Adjusted	Contact based	Occupation	Musa 2015	60.0
Nigeria	2014				33 - 46			Naive	Population based	Sex	Fawole 2016	40.0
Sierra Leone	Dec 2013 - 25 Nov 2014	31						Unspecified	Community based		Helleringer 2015	100.0
Sierra Leone	1 May 2014 - 31 Jan 2015	69						Unspecified	Hospital based	Occupation	Senga 2016	85.7
Sierra Leone	1 May 2014 - 31 Jan 2015	74						Unspecified	Hospital based	Occupation	Senga 2016	85.7
Sierra Leone	12 May 2014 - 13 Nov 2015				19 - 46			Unspecified		Other, Time	Chen 2021	71.4
Sierra Leone	19 May 2014 - 11 Jan 2015	51				12 - 107	95% CI	Unspecified	Hospital based		Li 2020	0.0
Sierra Leone	23 May 2014 - 31 Jan 2015	74	2,536	3,422		73 - 76	95% CrI	Naive	Population based		Wong 2016	75.0
Sierra Leone	25 May 2014 - 18 Jun 2014	74	64	87	33 - 94			Adjusted	Hospital based	Age, Other, Region, Sex	Schieffelin 2014	100.0
Sierra Leone	27 May 2014 - 21 Dec 2014				24 - 67			Adjusted	Population based	Time	Siettos 2015	14.3
Sierra Leone	28 May 2014 - 31 Dec 2015	46						Unspecified		Other, Time	Potluri 2020	100.0
Sierra Leone	May 2014 - Apr 2015	94	29	31		79 - 99	95% CI	Unspecified	Household based		Caleo 2018	100.0
Sierra Leone	1 Jun 2014 - 7 Nov 2015	37	1,816	4,954	25 - 73			Naive	Population based	Age	Lamunu 2017	40.0
Sierra Leone	23 Jun 2014 - 5 Oct 2014	53	259	488		49 - 58	95% CI	Unspecified	Hospital based	Occupation	Dallatomasina 2015	85.7
Sierra Leone	26 Jun 2014 - 12 Oct 2014	51	270	525	24 - 85			Adjusted	Hospital based	Occupation, Other, Region, Time	Fitzpatrick 2015	80.0
Sierra Leone	26 Jun 2014 - 31 Dec 2014	57	52	91	46 - 82			Unspecified	Hospital based	Age, Other, Region, Sex	Shah 2016	100.0
Sierra Leone	Jun 2014 - Apr 2015				0 - 38			Unspecified	Hospital based	Age, Other, Sex	Kangbai 2019	85.7
Sierra Leone	Jun 2014 - Apr 2015	26	279	1,077	1 - 100			Adjusted	Hospital based	Age, Occupation, Other, Sex, Symptoms	Kangbai 2020	100.0
Sierra Leone	Jun 2014 - Apr 2015	26	248	938	3 - 100			Adjusted	Hospital based	Age, Occupation, Other, Sex, Symptoms	Kangbai 2020 (b)	80.0
Sierra Leone	1 Jul 2014 - 30 Jun 2015	24	93	454	0 - 40			Unspecified	Population based	Region	Miglietta 2019	28.6
Sierra Leone	Jul 2014 - Nov 2014	86	42	49		73 - 94	95% CI	Naive	Population based		Ajelli 2015	71.4
Sierra Leone	14 Aug 2014 - 31 Mar 2015	57	160	282	0 - 100	51 - 63	95% CI	Adjusted	Hospital based	Age, Other, Region, Sex, Symptoms, Time	Fitzgerald 2016	80.0
Sierra Leone	12 Sep 2014 - 23 Feb 2015	44	110	249				Naive	Hospital based	Age, Other, Region, Sex, Symptoms	Theocharopoulos 2017	60.0
Sierra Leone	13 Sep 2014 - 26 Nov 2014	48	99	205	10 - 100			Adjusted	Hospital based	Age, Occupation, Other, Region, Sex	Kangbai 2021	80.0
Sierra Leone	Sep 2014 - Jan 2015	66	142	216				Adjusted	Community based	Other	Crowe 2016	85.7
Sierra Leone	1 Oct 2014 - 21 Mar 2015	49	139	285				Naive	Hospital based		Ji 2016	100.0
Sierra Leone	1 Oct 2014 - 14 Nov 2014	69						Unspecified	Hospital based	Age	Qin 2015	42.9
Sierra Leone	1 Oct 2014 - 9 Nov 2014	60	51	85				Unspecified	Hospital based		Yan 2015	100.0
Sierra Leone	11 Oct 2014 - 9 Dec 2014	51	38	74				Unspecified	Population based		DeSilva 2019	40.0
Sierra Leone	Oct 2014 - Mar 2015	73	98	134				Adjusted	Hospital based	Sex	Li 2016 (b)	100.0
Sierra Leone	Oct 2014 - Apr 2015	66	94	142	55 - 74	58 - 74	95% CI	Unspecified	Population based	Age, Sex	Muoghalu 2017	100.0
Sierra Leone	15 Nov 2014 - 18 Jan 2015	55	76	139	9 - 93			Naive	Hospital based	Age, Other, Sex, Symptoms	Xu 2016	100.0
Sierra Leone	1 Dec 2014 - 28 Feb 2015	64	32	50				Unspecified	Community based	Level of exposure	Stehling-Ariza 2016	100.0
Sierra Leone	1 Dec 2014 - 28 Feb 2015	63	27	43				Unspecified	Community based	Level of exposure	Stehling-Ariza 2016	100.0
Sierra Leone	8 Dec 2014 - 9 Jan 2015	37	55	150	24 - 40			Unspecified	Hospital based	Age, Other, Sex	Hunt 2015	71.4

Country	Survey date	Central estimate	Deaths	Cases	Central range	Uncertainty	Uncertainty type	Adjustment	Population Sample	Disaggregated by	Article	QA score (%)
Sierra Leone	12 Dec 2014 - 14 Mar 2015	46						Unspecified	Hospital based	Age	Rudolf 2017	60.0
Sierra Leone	12 Dec 2014 - 14 Mar 2015	67						Unspecified	Hospital based	Age	Rudolf 2017	60.0
Sierra Leone	13 Dec 2014 - 20 Apr 2015	50	50	101				Naive	Hospital based		Lanini 2015	100.0
Sierra Leone	19 Dec 2014 - 17 Feb 2015	58	18	31	35 - 86			Unspecified	Hospital based	Sex	Haaskjold 2016	85.7
Sierra Leone	21 Dec 2014 - 15 Aug 2015				10 - 39			Unspecified		Time	Siettos 2016	42.9
Sierra Leone	Dec 2014 - Jan 2015	78	28	36		61 - 90	95% CI	Naive	Unspecified		Richardson 2016	80.0
Sierra Leone	2014	75						Unspecified	Unspecified		Rivers 2014	42.9
Sierra Leone	13 Jan 2015 - 5 Apr 2015	57	8	14				Adjusted	Community based		Li 2016	100.0
Sierra Leone	16 Jan 2015 - 10 Mar 2015	47	29	62				Adjusted	Other		Jiang 2017	80.0
Sierra Leone	Jun 2015 - Jan 2016	58	227	395				Naive	Household based		Bower 2016	14.3
**DRC, 2014**												
DRC	26 Jul 2014 - 7 Oct 2014	74						Unspecified	Community based		Maganga 2014	28.6
DRC	13 Aug 2014 - 8 Sep 2014	62	23	37	62 - 68			Adjusted	Unspecified	Other	Li 2019	71.4
DRC	28 Aug 2014 - 8 Nov 2014	48	12	25				Unspecified	Hospital based		Nanclares 2016	80.0
**DRC, 2017**												
DRC	27 Mar 2017 - 1 Jul 2017	50	4	8				Naive			Nsio 2019	100.0
**DRC, 2018**												
DRC	4 Apr 2018 - 27 May 2018	52	25	48				Naive	Community based		Kelly 2019	28.6
DRC	5 Apr 2018 - 30 May 2018	50	25	50	36 - 64	36 - 64	95% CI	Naive	Unspecified		Ahuka-Mundeke 2018	100.0
DRC	5 Apr 2018 - 30 May 2018	56	25	50	39 - 72	39 - 72	95% CI	Adjusted	Unspecified		Ahuka-Mundeke 2018	100.0
**DRC, 2018-2020**												
DRC	Jul 2018 - May 2020	62	61	579	3 - 94			Unspecified	Hospital based	Age, Occupation	Baller 2022	0.0
DRC	5 Aug 2018 - 2 Feb 2020				50 - 80			Unspecified	Hospital based	Time	Potluri 2022	71.4
DRC	5 Aug 2018 - 2 Feb 2020				50 - 80			Unspecified	Community based	Time	Potluri 2022	71.4
DRC	7 Dec 2018 - 29 Jan 2020	63	157	248				Adjusted	Hospital based		Rupani 2022	80.0
DRC	7 Dec 2018 - 29 Jan 2020	25	34	137				Adjusted	Hospital based		Rupani 2022	80.0
DRC	30 Mar 2019 - 3 Aug 2019	55	117	213				Adjusted	Hospital based		Kasereka 2020	100.0
DRC	30 Mar 2019 - 3 Aug 2019	23	10	44				Adjusted	Hospital based		Kasereka 2020	100.0
DRC	24 Apr 2019 - 14 Oct 2019	45	139	307	41 - 71			Adjusted	Hospital based	Age	Kjaldgaard 2022	100.0
DRC	12 Jun 2019 - 18 May 2020	12	4	32				Unspecified	Other		Keita 2023	28.6
DRC	12 Jun 2019 - 18 May 2020	48	42	87				Unspecified	Community based		Keita 2023	28.6
Uganda	18 Sep 2020 - Dec 2000	71	12	17				Unspecified	Contact based		Francesconi 2003	57.1
Uganda	18 Sep 2020 - Dec 2000	100	10	10				Unspecified	Contact based		Francesconi 2003	57.1
**Multiple outbreaks**												
DRC	Aug 1976 - Nov 2012	79	526		44 - 96	76 - 82	95% CI	Adjusted	Unspecified	Other	Rosello 2015	100.0
Multi-country: Africa (n = 7)	1976 - 2014	66	1,595	2,411				Unspecified		Region, Time	Shultz 2016	42.9

4.5.2 Risk factors associated with severity

Table S22: A) Risk factors associated with death, B) Protective factors associated with recovery. Total is the number of parameters extracted for each risk/protective factor.

A

Risk Factor for Death	Significant			Not significant			Unspecified significance			Total
	Adjusted	Not adjusted	Unspecified	Adjusted	Not adjusted	Unspecified	Adjusted	Not adjusted	Unspecified	
Age	18	12	11	9	12	4	1	1	0	68
Sex	3	5	2	14	14	7	1	1	0	47
Occupation	1	1	1	6	5	2	0	0	1	17
Hospitalisation	2	2	2	1	0	0	0	0	0	7
Close contact	1	1	0	1	2	0	0	0	0	5
Comorbidity	0	0	0	0	4	1	0	0	0	5
Funeral	1	0	0	0	2	0	0	0	0	3
Household contact	0	0	0	2	1	0	0	0	0	3
Non-household contact	0	0	0	2	0	0	0	0	0	2
Other	26	22	11	21	22	9	2	1	0	114

B

Protective Factor for Recovery	Significant			Not significant			Total
	Adjusted	Not adjusted	Unspecified	Adjusted	Not adjusted	Unspecified	
Age	1	1	0	0	1	1	4
Sex	0	0	0	1	1	1	3
Hospitalisation	0	0	1	0	0	0	1
Other	1	2	0	1	1	0	5

4.6 Mutation rates

Table S23: Mutation rates

Country	Survey date	Central estimate	Central range	Central type	Uncertainty	Uncertainty type	Genome site	Population Sample	Sample size	Disaggregated by	Species	Article	QA score (%)
**Evolutionary rate (Substitutions/site/year (10^{-4}))**													
DRC	May 2007 - Feb 2009		1.5 - 7.5	Other/Unspecified			Whole genome, Glycoprotein, Nucleoprotein	Other			Zaire	Grard 2011	57.1
DRC, Guinea, Liberia, Sierra Leone	2013 - 2016	4.7	1 - 10	Mean	3.4 - 5.7	95% CrI	Whole genome, Partial sequences		85	Other	Zaire	Li 2019	71.4
Guinea, Liberia, Sierra Leone	2013 - 2015	7.3		Other/Unspecified			Glycoprotein		600		Zaire	Dudas 2019	71.4
Guinea, Liberia, Sierra Leone	2013 - 2015	8.2		Other/Unspecified			Whole genome		600		Zaire	Dudas 2019	71.4
Guinea, Mali, Sierra Leone	Dec 2013 - Jan 2015	14.2		Mean	12.2 - 16.2	95% CrI	Whole genome		179		Zaire	Carroll 2015	57.1
Guinea, Sierra Leone	2014 - 2015	12.5		Mean			Partial genome		318		Zaire	Park 2015	100.0
Sierra Leone	May 2014 - Sep 2015	9.6	5 - 9.63	Mean	8.6 - 10.6	95% CrI	Whole genome			Other	Zaire	Whitmer 2018	100.0
Guinea	12 Feb 2021 - 4 Mar 2021	11		Other/Unspecified			Whole genome, Partial genome		12		Zaire	Keita 2021	71.4
DRC	Aug 1976 - Aug 2018		2.4 - 8.6	Mean			Whole genome	Population based	15	Other, Time	Zaire	Mbala-Kingebeni 2019	100.0
Multi-country: Africa (n = 5)	1976 - 2014	10.75		Median	9.32 - 12.2	HPDI 95%	Glycoprotein		65	Other	Zaire	Azarian 2015	100.0
Multi-country: Africa, Asia, Europe, USA (n = 9)	1976 - 2008	10.93	7.66 - 13.94	Other/Unspecified	0.52 - 24.61	HPDI 95%	Glycoprotein				All species	Li 2014	100.0
Multi-country: Africa, Asia, Europe, USA (n = 9)	1976 - 2008	23.14		Other/Unspecified	16.49 - 31.66	HPDI 95%	Polymerase (L)				All species	Li 2014	100.0
**Mutation rate (Substitutions/site/year (10^{-4}))**													
Liberia	Sep 2014 - Feb 2015	9.17		Other/Unspecified	0.523	Other	Whole genome	Hospital based	25	Other	Zaire	Kugelman 2015	28.6
Liberia	Sep 2014 - Feb 2015		9.44 - 15.67	Other/Unspecified			Whole genome	Hospital based	25	Other	Zaire	Kugelman 2015	28.6
**Substitution rate (Substitutions/site/year (10^{-4}))**													
DRC	3 Nov 2007 - 10 Sep 2012	8.4		Mean	1.1 - 21	95% CrI	Whole genome		12		Bundibugyo	Hulseberg 2021	100.0
Guinea	Feb 2014 - Unspecified	10.7		Mean			Glycoprotein				Zaire	Dudas 2014	60.0
Guinea	Unspecified	2		Mean	1 - 3	HPDI 95%	Partial genome		49		All species	Membrebe 2019	57.1
Guinea, Mali, Sierra Leone	Mar 2014 - Dec 2014		8.7 - 9.1	Mean	6.8 - 11	95% CrI	Whole genome	Hospital based	195		Zaire	Simon-Loriere 2015	71.4
Guinea, Mali, Sierra Leone	2014	13		Mean			Whole genome		106		Zaire	Hoenen 2015	83.3
Guinea, Sierra Leone	28 Sep 2014 - 11 Nov 2014	12.3		Other/Unspecified	10.4 - 14.1	HPDI 95%	Whole genome	Community based	175		Zaire	Tong 2015	42.9
DRC	8 May 2018 - 24 Jul 2018	36		Mean	12.2 - 59.9	HPDI 95%	Whole genome		15		Zaire	Mbala-Kingebeni 2019 (b)	71.4
Multi-country: Africa (n = 5)	15 Aug 1976 - 4 Jun 2018	6.9		Mean	5.5 - 8.3	HPDI 95%	Whole genome		50		Zaire	Mbala-Kingebeni 2019 (b)	71.4
Multi-country: Africa, Asia, Europe, USA (n = 6)	1976 - 1995	0.36		Mean	0.109	Standard Error	Glycoprotein				Unspecified	Suzuki 1997	42.9
DRC	Unspecified	1.4		Mean	0.473 - 2.45	95% CrI	Whole genome				Zaire	Vrancken 2020	85.7

4.7 Transmission Models

Table S24: Summary of EVD model types.

Model type	Total
Compartmental	210
Other or combination	49
Branching process	19
Agent/Individual based	17

Table S25: Summary of EVD model assumptions. Total is the number of models with this assumption extracted (assumptions were not extracted for all models).

Assumptions	Total
**Compartmental**	
Homogeneous mixing	83
Heterogeneity in transmission rates between groups	43
Heterogeneity in transmission rates over time	42
Latent period the same as incubation period	27
**Other or combination**	
Heterogeneity in transmission rates between groups	15
Heterogeneity in transmission rates over time	11
Homogeneous mixing	9
Latent period the same as incubation period	5
Age dependent susceptibility	1
**Branching process**	
Heterogeneity in transmission rates over time	4
Heterogeneity in transmission rates between groups	3
Homogeneous mixing	1
**Agent/Individual based**	
Heterogeneity in transmission rates between groups	9
Heterogeneity in transmission rates over time	4
Latent period the same as incubation period	3
Age dependent susceptibility	2

Table S26: Models overview. * = Zaire, ** = Bundibugyo, *** = Bundibugyo, Sudan, Taï Forest and Zaire

Stochastic or Deterministic	Theoretical model	Interventions	Code available	Assumptions	Article	QA score (%)
**Agent/Individual based**						
Stochastic	Yes	Behaviour changes, Contact tracing, Hospitals, Safe burials, Treatment Centres		Age dependent susceptibility, Heterogeneity in transmission rates between groups	Ajelli 2018	100.00
Stochastic	Yes	Vaccination		Heterogeneity in transmission rates between groups, Unspecified	Bisanzio 2023	100.00
Stochastic				Heterogeneity in transmission rates between groups and over time	Cope 2014	100.00
Stochastic		Hospitals, Other		Unspecified	Fadikar 2018	100.00
Stochastic			Yes	Heterogeneity in transmission rates between groups, Latent period the same as incubation period	Kahn 2020	100.00
Stochastic		Other, Safe burials, Treatment Centres		Heterogeneity in transmission rates between groups	Merler 2015	100.00
Stochastic		Vaccination		Heterogeneity in transmission rates between groups	Merler 2016	100.00
Stochastic		Contact tracing, Hospitals, Safe burials		Heterogeneity in transmission rates over time	Rizzo 2016	100.00
Stochastic		Hospitals		Unspecified	Gomes 2014	85.71
Stochastic				Unspecified	Gomez-Barroso 2017	80.00
Stochastic		Contact tracing, Hospitals, Safe burials, Treatment Centres, Vaccination		Age dependent susceptibility, Heterogeneity in transmission rates between groups	Ajelli 2016	71.43
Stochastic	Yes			Heterogeneity in transmission rates between groups	Kustudic 2021	50.00
Stochastic				Unspecified	Fallah 2015	42.86
Stochastic		Other, Safe burials		Heterogeneity in transmission rates over time, Latent period the same as incubation period	Siettos 2016	42.86
Stochastic				Heterogeneity in transmission rates between groups and over time, Latent period the same as incubation period	Uekermann 2019	28.57
Stochastic	Yes	Behaviour changes, Hospitals, Other, Quarantine, Safe burials		Unspecified	Venkatramanan 2018	25.00
				Unspecified	Khaleque 2017	0.00
**Branching process**						
Stochastic				Unspecified	Djaafara 2021	100.00
Stochastic		Quarantine, Vaccination	Yes	Heterogeneity in transmission rates between groups and over time	Green 2022	100.00
Stochastic				Unspecified	Holbrook 2022	100.00
Stochastic			Yes	Heterogeneity in transmission rates over time	Kelly 2019 (b)	100.00
Stochastic				Unspecified	Nouvellet 2018	100.00
Stochastic		Other, Quarantine	Yes	Heterogeneity in transmission rates between groups	Yamin 2016	100.00
Stochastic				Unspecified	Nishiura 2014	75.00
Stochastic		Quarantine, Safe burials		Unspecified	Fang 2016	71.43
Stochastic		Vaccination		Unspecified	Kucharski 2016	71.43
Stochastic				Unspecified	Toth 2015	71.43
Stochastic		Hospitals, Safe burials		Heterogeneity in transmission rates between groups	Drake 2015	57.14
Stochastic				Heterogeneity in transmission rates over time	Zhang 2022	57.14
Stochastic			Yes	Unspecified	Lee 2022	50.00
Stochastic				Unspecified	Choi 2019**	42.86
Stochastic				Unspecified	Jombart 2020	42.86
Stochastic		Vaccination		Heterogeneity in transmission rates over time	Worden 2019	42.86
Stochastic		Vaccination		Homogeneous mixing	Kelly 2019	28.57
Stochastic				Unspecified	Champredon 2018	25.00
Stochastic	Yes		Yes	Unspecified	Park 2019	25.00
**Compartmental**						
Deterministic	Yes			Homogeneous mixing, Latent period the same as incubation period, Unspecified	Almocera 2019	100.00
Deterministic	Yes			Heterogeneity in transmission rates between groups	Altaf 2019	100.00
Deterministic	Yes	Other, Vaccination		Homogeneous mixing	Anguelov 2020	100.00
Stochastic				Homogeneous mixing	Asher 2018	100.00
Deterministic	Yes			Homogeneous mixing	Baldassi 2016*	100.00

Stochastic or Deterministic	Theoretical model	Interventions	Code available	Assumptions	Article	QA score (%)
Deterministic	Yes			Homogeneous mixing, Latent period the same as incubation period	Berge 2016	100.00
Deterministic	Yes			Homogeneous mixing, Latent period the same as incubation period	Berge 2018	100.00
Deterministic	Yes	Contact tracing, Quarantine		Homogeneous mixing	Berge 2018 (b)	100.00
Deterministic	Yes			Heterogeneity in transmission rates between groups, Homogeneous mixing	Berge 2018 (c)	100.00
Deterministic	Yes			Heterogeneity in transmission rates between groups, Homogeneous mixing	Berge 2018 (c)	100.00
Deterministic	Yes	Behaviour changes, Other, Vaccination		Homogeneous mixing	Berge 2018 (d)	100.00
Deterministic	Yes	Vaccination		Heterogeneity in transmission rates between groups, Homogeneous mixing	Bhunu 2016	100.00
Deterministic	Yes	Hospitals, Quarantine, Safe burials		Heterogeneity in transmission rates between groups and over time	Blackwood 2016	100.00
Deterministic		Vaccination		Heterogeneity in transmission rates between groups, Latent period the same as incubation period	Bodine 2018	100.00
Stochastic		Hospitals, Safe burials, Treatment Centres		Heterogeneity in transmission rates over time	Camacho 2015	100.00
Deterministic				Homogeneous mixing	Chapwanya 2022	100.00
	Yes	Treatment, Vaccination		Heterogeneity in transmission rates between groups and over time	De la Sen 2017 (b)	100.00
Deterministic	Yes	Safe burials, Treatment, Vaccination		Unspecified	De la Sen 2019	100.00
Stochastic				Homogeneous mixing	Frasso 2016	100.00
Stochastic		Hospitals		Heterogeneity in transmission rates over time	Funk 2017	100.00
Stochastic			Yes	Heterogeneity in transmission rates over time	Funk 2018	100.00
Stochastic			Yes	Heterogeneity in transmission rates over time	Funk 2019	100.00
Stochastic	Yes	Other, Vaccination	Yes	Unspecified	Getz 2015	100.00
Deterministic	Yes	Hospitals, Quarantine, Treatment		Unspecified	Gutfraind 2015	100.00
Stochastic	Yes			Homogeneous mixing	Kamara 2020	100.00
Stochastic		Behaviour changes, Hospitals, Quarantine		Heterogeneity in transmission rates between groups	Ko 2019	100.00
Stochastic			Yes	Unspecified	Kramer 2016	100.00
Stochastic		Treatment Centres		Heterogeneity in transmission rates over time	Kucharski 2015	100.00
Deterministic				Unspecified	Leander 2016	100.00
Deterministic		Vaccination		Heterogeneity in transmission rates between groups and over time, Homogeneous mixing, Latent period the same as incubation period	Lee 2019	100.00
Stochastic		Hospitals, Safe burials		Homogeneous mixing	Legrand 2007	100.00
Stochastic	Yes		Yes	Homogeneous mixing	Lekone 2006	100.00
Deterministic		Other, Quarantine, Treatment, Vaccination		Homogeneous mixing	Mbah 2022	100.00
Deterministic	Yes	Behaviour changes, Safe burials		Unspecified	Mhlanga 2019	100.00
	Yes			Unspecified	Nazir 2020	100.00
Stochastic	Yes			Homogeneous mixing	Nieddu 2017	100.00
Deterministic	Yes	Other		Unspecified	Njankou 2022	100.00
Deterministic	Yes			Heterogeneity in transmission rates between groups	Njankou 2023	100.00
Stochastic		Hospitals, Vaccination		Heterogeneity in transmission rates between groups and over time, Homogeneous mixing	Potluri 2020	100.00
Stochastic				Heterogeneity in transmission rates between groups and over time, Homogeneous mixing, Latent period the same as incubation period	Riad 2019	100.00
Deterministic		Vaccination		Heterogeneity in transmission rates between groups and over time	Robert 2019	100.00
Deterministic			Yes	Heterogeneity in transmission rates over time, Homogeneous mixing	Santermans 2016	100.00
Deterministic	Yes			Heterogeneity in transmission rates between groups	Seidu 2020	100.00
Stochastic				Unspecified	Shakiba 2021	100.00
Deterministic	Yes	Other		Unspecified	Siewe 2020	100.00
Deterministic			Yes	Unspecified	Stadler 2014	100.00
Deterministic	Yes			Latent period the same as incubation period	Verma 2019	100.00
	Yes	Hospitals, Safe burials		Unspecified	Wang 2017	100.00
Deterministic				Unspecified	Wiratsudakul 2016	100.00

Stochastic or Deterministic	Theoretical model	Interventions	Code available	Assumptions	Article	QA score (%)
		Treatment		Unspecified	Zaman 2009	100.00
Deterministic			Yes	Homogeneous mixing	Abbate 2016	85.71
Deterministic	Yes	Behaviour changes	Yes	Homogeneous mixing	Abbate 2016	85.71
Deterministic		Hospitals, Safe burials		Heterogeneity in transmission rates between groups, Homogeneous mixing	Diaz 2018	85.71
Stochastic				Heterogeneity in transmission rates between groups	Enanoria 2015	85.71
Deterministic		Other, Treatment Centres		Homogeneous mixing	Lewnard 2014	85.71
Stochastic				Unspecified	Luo 2019	85.71
Stochastic			Yes	Heterogeneity in transmission rates between groups and over time	Muleia 2021	85.71
Deterministic		Safe burials		Latent period the same as incubation period	Sofonea 2018	85.71
Stochastic		Quarantine, Safe burials, Treatment		Heterogeneity in transmission rates over time	Volz 2014	85.71
Deterministic		Quarantine, Safe burials, Treatment		Heterogeneity in transmission rates over time	Volz 2014	85.71
Deterministic		Quarantine, Safe burials, Treatment		Unspecified	Volz 2014	85.71
		Hospitals, Safe burials		Unspecified	Xia 2015	85.71
Deterministic				Heterogeneity in transmission rates over time, Homogeneous mixing	Yang 2015	85.71
Stochastic				Unspecified	Gomez-Barroso 2017	80.00
Deterministic	Yes	Quarantine		Unspecified	Abbasi 2020	75.00
Deterministic	Yes	Other		Homogeneous mixing	Agusto 2016*	75.00
Deterministic	Yes	Hospitals, Quarantine, Vaccination		Heterogeneity in transmission rates between groups, Homogeneous mixing	Ahmad 2016*	75.00
Deterministic & Stochastic				Unspecified	Atkins 2016	75.00
Deterministic	Yes	Vaccination		Homogeneous mixing	Brettin 2018	75.00
				Unspecified	Burghardt 2016	75.00
		Other		Heterogeneity in transmission rates between groups and over time, Latent period the same as incubation period	Buyuktahtakin 2018	75.00
	Yes	Hospitals		Heterogeneity in transmission rates between groups	Dautel 2021	75.00
Deterministic		Behaviour changes		Heterogeneity in transmission rates over time	Djiomba Njankou 2022	75.00
Deterministic	Yes			Unspecified	Dokuyucu 2020	75.00
Deterministic	Yes			Homogeneous mixing	El Rhoubari 2019	75.00
Deterministic	Yes			Unspecified	Farman 2022 (b)	75.00
Stochastic				Heterogeneity in transmission rates over time	Getz 2018	75.00
Deterministic				Heterogeneity in transmission rates over time	Getz 2018	75.00
Deterministic				Heterogeneity in transmission rates over time	Getz 2018	75.00
Stochastic				Heterogeneity in transmission rates over time	Getz 2018	75.00
Deterministic	Yes	Behaviour changes, Other, Safe burials		Unspecified	Guo 2016	75.00
Deterministic & Stochastic	Yes			Unspecified	Kamara 2021	75.00
Deterministic	Yes	Quarantine		Heterogeneity in transmission rates between groups	Ngwa 2016	75.00
Deterministic	Yes	Other, Quarantine, Treatment, Vaccination		Heterogeneity in transmission rates between groups	Ojoma 2021	75.00
Stochastic	Yes	Contact tracing		Heterogeneity in transmission rates between groups	Shahtori 2018	75.00
Deterministic	Yes			Latent period the same as incubation period	Tahir 2019	75.00
Stochastic		Behaviour changes		Unspecified	Vinson 2016	75.00
		Safe burial, Unspecified		Homogeneous mixing	Weitz 2015	75.00
Deterministic		Contact tracing, Other, Quarantine		Heterogeneity in transmission rates over time, Homogeneous mixing, Latent period the same as incubation period, Unspecified	Althaus 2015	71.43
Deterministic		Contact tracing, Other, Quarantine, Safe burials, Treatment	Yes	Heterogeneity in transmission rates over time, Homogeneous mixing, Latent period the same as incubation period, Unspecified	Althaus 2015 (b)	71.43
Deterministic & Stochastic		Behaviour changes, Hospitals, Safe burials		Heterogeneity in transmission rates between groups and over time, Homogeneous mixing	Camacho 2014	71.43
Deterministic		Other, Quarantine, Treatment Centres	Yes	Heterogeneity in transmission rates between groups	D'Silva 2017*	71.43
Stochastic		Other, Quarantine, Treatment Centres	Yes	Heterogeneity in transmission rates between groups	D'Silva 2017*	71.43

Stochastic or Deterministic	Theoretical model	Interventions	Code available	Assumptions	Article	QA score (%)
Stochastic				Heterogeneity in transmission rates between groups	Fasina 2014*	71.43
Deterministic				Heterogeneity in transmission rates over time	Getz 2019	71.43
Deterministic		Other, Quarantine	Yes	Heterogeneity in transmission rates over time, Homogeneous mixing, Latent period the same as incubation period	Hart 2019	71.43
Stochastic		Vaccination		Unspecified	Potluri 2022	71.43
		Other, Quarantine, Safe burials, Vaccination		Heterogeneity in transmission rates between groups and over time	Shen 2015	71.43
Stochastic		Contact tracing		Unspecified	Webb 2015*	71.43
Deterministic		Behaviour changes, Hospitals, Other, Safe burials		Heterogeneity in transmission rates between groups, Homogeneous mixing	Agusto 2015*	57.14
Deterministic				Unspecified	Li 2017 (b)	57.14
Deterministic & Stochastic				Homogeneous mixing	Mouanguissa 2021	57.14
Stochastic				Homogeneous mixing	Ndanguza 2017	57.14
Deterministic		Treatment Centres		Homogeneous mixing	Njankou 2018	57.14
Stochastic		Hospitals, Behaviour changes, Safe burials		Heterogeneity in transmission rates between groups, Latent period the same as incubation period	Valdez 2015	57.14
		Hospitals		Unspecified	Webb 2016	57.14
Deterministic		Quarantine, Treatment		Homogeneous mixing, Latent period the same as incubation period, Heterogeneity in transmission rates over time	White 2015	57.14
Deterministic		Contact tracing, Hospitals, Other, Vaccination		Heterogeneity in transmission rates over time, Latent period the same as incubation period	Xie 2019	57.14
Deterministic		Other, Safe burials		Unspecified	Agbomola 2022	50.00
Deterministic	Yes	Vaccination		Homogeneous mixing, Latent period the same as incubation period	Ahmad 2020	50.00
Deterministic	Yes	Quarantine		Homogeneous mixing	Ahmad 2021	50.00
Stochastic	Yes	Contact tracing, Other, Quarantine, Safe burials		Unspecified	Bouba 2023	50.00
Stochastic				Unspecified	Brown 2016	50.00
Deterministic	Yes		Yes	Homogeneous mixing	Cheema 2021	50.00
Deterministic		Hospitals, Quarantine		Homogeneous mixing, Latent period the same as incubation period	Chinyoka 2021	50.00
Deterministic	Yes	Treatment, Vaccination		Homogeneous mixing	Chukwu 2020	50.00
Deterministic		Safe burials, Treatment Centres, Vaccination		Heterogeneity in transmission rates between groups and over time	Diakite 2016*	50.00
Deterministic		Quarantine		Heterogeneity in transmission rates between groups, Homogeneous mixing, Latent period the same as incubation period	Diakite 2016	50.00
Deterministic	Yes	Hospitals, Safe burials		Unspecified	Gaffey 2018	50.00
Deterministic		Hospitals, Treatment, Vaccination		Latent period the same as incubation period	Jiang 2017 (b)	50.00
Deterministic		Hospitals, Other, Safe burials		Homogeneous mixing	Juga 2023	50.00
Deterministic	Yes			Unspecified	Lopez 2016	50.00
Deterministic	Yes	Quarantine		Homogeneous mixing	Nisar 2023	50.00
Deterministic	Yes	Vaccination		Homogeneous mixing, Latent period the same as incubation period	Obeng-Kusi 2021	50.00
Deterministic				Unspecified	Ord 2018	50.00
Deterministic	Yes	Behaviour changes, Other, Vaccination		Homogeneous mixing	Rachah 2016	50.00
Deterministic	Yes	Treatment, Vaccination		Homogeneous mixing	Rachah 2016 (b)	50.00
Deterministic	Yes	Vaccination		Homogeneous mixing	Rachah 2017	50.00
	Yes			Unspecified	Rafiq 2020	50.00
Deterministic	Yes	Behaviour changes, Hospitals, Safe burials		Heterogeneity in transmission rates over time, Homogeneous mixing	Roy 2017	50.00
Deterministic	Yes	Behaviour changes, Hospitals, Other, Vaccination		Homogeneous mixing, Latent period the same as incubation period	Seck 2022	50.00
Deterministic				Unspecified	Singh 2023	50.00
Deterministic				Heterogeneity in transmission rates over time	Smirnova 2019	50.00
Deterministic	Yes			Homogeneous mixing	Srivastava 2020	50.00
Deterministic	Yes			Unspecified	Srivastava 2020 (b)	50.00
Deterministic	Yes			Unspecified	Srivastava 2021	50.00
Deterministic	Yes	Vaccination		Homogeneous mixing	Tahir 2018	50.00
Deterministic	Yes			Homogeneous mixing, Latent period the same as incubation period	Tandon 2018	50.00

Stochastic or Deterministic	Theoretical model	Interventions	Code available	Assumptions	Article	QA score (%)
Deterministic	Yes			Unspecified	Telionis 2020	50.00
Deterministic	Yes		Yes	Homogeneous mixing	Tsanou 2017	50.00
Deterministic & Stochastic		Quarantine, Vaccination		Homogeneous mixing	Tulu 2017 (b)	50.00
Deterministic	Yes			Homogeneous mixing, Latent period the same as incubation period	Tulu 2017 (c)	50.00
Stochastic	Yes			Unspecified	Wang 2019	50.00
Stochastic	Yes	Treatment Centres		Heterogeneity in transmission rates between groups	Yin 2021	50.00
Deterministic	Yes	Behaviour changes, Other, Treatment		Heterogeneity in transmission rates between groups	Zakary 2017	50.00
Deterministic		Other		Heterogeneity in transmission rates over time, Homogeneous mixing, Latent period the same as incubation period, Unspecified	Althaus 2014	42.86
Deterministic		Other, Quarantine, Safe burials, Treatment Centres		Heterogeneity in transmission rates over time, Homogeneous mixing, Latent period the same as incubation period	Barbarossa 2015	42.86
Stochastic		Vaccination		Unspecified	Camacho 2015 (b)	42.86
				Unspecified	Evans 2015	42.86
Deterministic		Quarantine, Safe burials		Homogeneous mixing	Imran 2017	42.86
Deterministic		Other	Yes	Homogeneous mixing	Ivorra 2015	42.86
Deterministic		Treatment		Homogeneous mixing	Li 2015	42.86
Deterministic				Unspecified	Ndanguza 2013*	42.86
Stochastic		Hospitals, Safe burials		Heterogeneity in transmission rates between groups, Homogeneous mixing	Ponce 2019	42.86
Deterministic		Vaccination		Homogeneous mixing	Rachah 2018	42.86
Deterministic & Stochastic		Contact tracing, Other, Treatment		Homogeneous mixing, Latent period the same as incubation period	Rivers 2014	42.86
Deterministic			Yes	Heterogeneity in transmission rates over time	Stockdale 2021	42.86
Stochastic			Yes	Heterogeneity in transmission rates over time	Tang 2023	42.86
Stochastic			Yes	Heterogeneity in transmission rates over time	Vossler 2022	42.86
Deterministic				Unspecified	Wang 2015	42.86
Stochastic			Yes	Unspecified	Ward 2023	42.86
Stochastic		Other		Heterogeneity in transmission rates over time	Chowell 2004	28.57
Deterministic		Hospitals		Unspecified	Juga 2020	28.57
Deterministic		Behaviour changes, Hospitals, Other		Homogeneous mixing	Juga 2021	28.57
Deterministic		Quarantine		Heterogeneity in transmission rates between groups	Khan 2015	28.57
Deterministic		Other		Heterogeneity in transmission rates between groups and over time, Homogeneous mixing	Siewe 2020 (b)	28.57
Deterministic		Behaviour changes, Quarantine		Unspecified	Tadmon 2022	28.57
Deterministic				Unspecified	Area 2015*	25.00
Deterministic	Yes			Unspecified	Atangana 2014	25.00
Deterministic	Yes		Yes	Unspecified	Bachinsky 2013	25.00
Deterministic	Yes			Homogeneous mixing	Bhardwaj 2021	25.00
Stochastic				Unspecified	Champredon 2018	25.00
Deterministic	Yes	Contact tracing, Hospitals, Other, Quarantine		Unspecified	Chen 2014***	25.00
	Yes			Unspecified	Dike 2017	25.00
Deterministic	Yes			Homogeneous mixing	Emile Franc Doungmo 2016*	25.00
	Yes			Unspecified	Gong 2019	25.00
Deterministic	Yes	Other		Homogeneous mixing	Grigorieva 2015	25.00
Deterministic	Yes	Other, Quarantine		Homogeneous mixing	Grigorieva 2017	25.00
Deterministic	Yes	Hospitals, Other, Safe burials		Homogeneous mixing	Grigorieva 2018	25.00
Deterministic	Yes			Unspecified	Hasan 2023	25.00
Stochastic				Unspecified	Kaur 2021	25.00
Deterministic		Behaviour changes		Heterogeneity in transmission rates between groups	Levy 2017	25.00

Stochastic or Deterministic	Theoretical model	Interventions	Code available	Assumptions	Article	QA score (%)
Deterministic				Unspecified	Mubayi 2021	25.00
	Yes	Hospitals, Behaviour changes		Heterogeneity in transmission rates between groups	Shah 2019	25.00
Deterministic	Yes	Contact tracing, Hospitals, Quarantine, Safe burials, Vaccination		Unspecified	Tadmon 2022 (b)	25.00
Deterministic & Stochastic		Quarantine, Vaccination		Homogeneous mixing	Tulu 2017	25.00
Deterministic	Yes			Homogeneous mixing	Zafar 2021	25.00
Deterministic		Quarantine, Safe burials		Unspecified	Do 2016	14.29
Deterministic		Other		Heterogeneity in transmission rates between groups	Dong 2015	14.29
	Yes	Safe burials, Vaccination		Heterogeneity in transmission rates over time	Area 2018	0.00
Stochastic	Yes			Unspecified	Atangana 2021	0.00
Deterministic & Stochastic		Quarantine, Vaccination		Unspecified	Din 2022	0.00
	Yes	Quarantine		Unspecified	Farman 2022	0.00
	Yes			Unspecified	Koca 2018	0.00
		Quarantine, Treatment, Vaccination		Unspecified	Li 2015 (b)	0.00
Deterministic				Homogeneous mixing	Li 2020	0.00
	Yes	Quarantine, Vaccination		Unspecified	Liu 2019	0.00
Deterministic		Behaviour changes		Heterogeneity in transmission rates between groups and over time	Long 2018	0.00
Deterministic	Yes	Contact tracing, Quarantine		Homogeneous mixing	Madubueze 2018	0.00
Deterministic	Yes			Unspecified	Momani 2022	0.00
Deterministic	Yes	Other, Treatment, Vaccination		Unspecified	Okyere 2020	0.00
Deterministic		Hospitals, Quarantine, Safe burials		Unspecified	Ouemba Tasse 2022	0.00
Deterministic				Unspecified	Pan 2021	0.00
Deterministic	Yes	Vaccination		Homogeneous mixing	Rachah 2015	0.00
Stochastic	Yes			Heterogeneity in transmission rates between groups	Rashid 2022	0.00
Deterministic	Yes			Unspecified	Raza 2020	0.00
	Yes	Treatment		Unspecified	Shaikh 2023	0.00
Deterministic	Yes			Homogeneous mixing	Singh 2020	0.00
Deterministic				Heterogeneity in transmission rates between groups	Sivaraman 2022	0.00
Deterministic	Yes			Homogeneous mixing	Zhang 2020	0.00
**Other or combination**						
Deterministic	Yes	Quarantine		Unspecified	Adams 2016	100.00
Stochastic		Vaccination		Heterogeneity in transmission rates between groups	Chowell 2019 (b)	100.00
			Yes	Unspecified	De Maio 2018	100.00
Deterministic				Unspecified	De la Sen 2017	100.00
Deterministic	Yes			Homogeneous mixing	Feng 2016	100.00
Stochastic				Heterogeneity in transmission rates over time	Ganyani 2018	100.00
				Unspecified	Gustafson 2017	100.00
Stochastic	Yes			Unspecified	Jacobsen 2018	100.00
Stochastic			Yes	Heterogeneity in transmission rates between groups	Kiskowski 2014	100.00
Stochastic				Heterogeneity in transmission rates between groups	Kiskowski 2016	100.00
Stochastic			Yes	Heterogeneity in transmission rates between groups and over time	Kraemer 2019	100.00
Stochastic				Heterogeneity in transmission rates between groups and over time, Latent period the same as incubation period	Lau 2017 (b)	100.00
Stochastic		Behaviour changes		Heterogeneity in transmission rates over time, Homogeneous mixing	Lee 2019	100.00
Stochastic	Yes			Unspecified	Pell 2016	100.00
Deterministic			Yes	Heterogeneity in transmission rates between groups	Santermans 2016	100.00
Stochastic				Heterogeneity in transmission rates over time	Shaman 2014	100.00

Stochastic or Deterministic	Theoretical model	Interventions	Code available	Assumptions	Article	QA score (%)
Deterministic				Unspecified	Smirnova 2017	100.00
				Unspecified	Tuitea 2018	100.00
	Yes			Unspecified	Zabinski 2018	100.00
Stochastic		Contact tracing, Quarantine		Heterogeneity in transmission rates over time	Chan 2020	85.71
			Yes	Homogeneous mixing	Chowell 2019	85.71
Stochastic			Yes	Heterogeneity in transmission rates between groups and over time, Latent period the same as incubation period	Lau 2017	85.71
Stochastic				Homogeneous mixing	White 2007*	85.71
	Yes			Unspecified	Mangiarotti 2016*	75.00
Deterministic	Yes			Homogeneous mixing	Upadhyay 2016*	75.00
Deterministic		Other, Vaccination		Unspecified	Chen 2021	71.43
Stochastic		Behaviour changes	Yes	Unspecified	Pruyt 2015	71.43
				Unspecified	Saulnier 2017	71.43
				Unspecified	Taylor 2016	71.43
				Unspecified	Browne 2015	57.14
Stochastic				Heterogeneity in transmission rates between groups and over time	Martinez 2022	57.14
Stochastic				Heterogeneity in transmission rates over time	Park 2022	57.14
Stochastic			Yes	Homogeneous mixing	Vaughan 2019	57.14
Deterministic	Yes			Heterogeneity in transmission rates between groups	Ahmed 2023	50.00
Stochastic				Unspecified	Backer 2016	50.00
Stochastic				Heterogeneity in transmission rates between groups	Chen 2016	50.00
Stochastic	Yes	Vaccination	Yes	Age dependent susceptibility, Heterogeneity in transmission rates between groups	Rachah 2016 (b)	50.00
Deterministic	Yes			Unspecified	Yamazaki 2018	50.00
Stochastic				Unspecified	Burch 2017	42.86
Deterministic				Unspecified	Jombart 2020	42.86
Stochastic				Latent period the same as incubation period	Russo 2016	42.86
Deterministic				Heterogeneity in transmission rates between groups, Homogeneous mixing	Scarpino 2015	42.86
Deterministic		Vaccination		Homogeneous mixing, Latent period the same as incubation period, Unspecified	Fisman 2014	28.57
Deterministic				Homogeneous mixing, Latent period the same as incubation period, Unspecified	Fisman 2014 (b)	28.57
				Unspecified	Hsieh 2015	28.57
Stochastic	Yes	Behaviour changes		Heterogeneity in transmission rates between groups and over time	Halvorsen 2022	25.00
Deterministic	Yes	Contact tracing, Other, Quarantine, Safe burials		Heterogeneity in transmission rates between groups and over time	Ivorra 2020	25.00
Stochastic		Safe burials		Heterogeneity in transmission rates between groups	Siettos 2015*	14.29
Stochastic	Yes			Unspecified	Burkhead 2015	0.00

Table S27: Summary of EVD model's with code.

Article	QA score (%)	Purpose	Model type	Heath states	Uncertainty considered	Fitted?	Fitting Method	Data	Outbreak period	Spatial model	Spillover included	Language	Link	README?
Kahn 2020	100.00	Transmission	Agent/Individual based	Susceptible, exposed, and recovered	No	Yes	MLE	Incidence of cases	All	Yes	No	Python	https://github.com/rek160/Sierra-Leone-Cholera-Ebola	No
Yamin 2016	100.00	Transmission	Branching process	Incubation, early symptomatic, and late symptomatic	Yes	No			Middle	No	No	Mathematica	https://www.acpjournals.org/doi/suppl/10.7326/M14-2255/suppl_file/AIME201501060-00154_supplement6.pdf	No
Park 2019	25.00	Methodological development	Branching process	Not compartmental	No	No			Unspecified	No	No	R	https://zenodo.org/records/2532906	Yes
Green 2022	100.00	Transmission	Branching process	Not compartmental	Yes	Yes	Unclear	Incidence of cases	All	No	No	R	https://github.com/willgreen236/Heterogeneity_transmission	No
Ward 2023	42.86	Methodological development	Compartmental	Susceptible, exposed, infectious, recovered	Yes	Yes	MCMC	Incidence of cases	All	No	No	R	https://github.com/ceward18/BayesSEIR	Yes
Vossler 2022	42.86	Transmission	Compartmental	Susceptible, infectious, recovered	Yes	Yes	MCMC	Incidence of cases	All	No	No	R	https://doi.org/10.5281/zenodo.6104188	Yes
Tang 2023	42.86	Transmission	Compartmental		Yes	Yes	MCMC	Sequencing data	All	No	No	R	https://projecteuclid-org.iclibezp1.cc.ic.ac.uk/journals/supplementalcontent/10.1214/21-AOAS1583/aoas1583suppb.zip	Yes
Stockdale 2021	42.86	Transmission	Compartmental	Susceptible, exposed, infectious, recovered	No	Yes	MLE	Incidence of deaths	All	No	No	R, C++	https://github.com/jessicastockdale/PBLA	Yes
Santermans 2016	100.00	Transmission	Compartmental	Susceptible, exposed, infectious, recovered	Yes	Yes	Other	Incidence of cases AND deaths		Yes	No	R	https://zenodo.org/records/33126	Yes
Muleia 2021	85.71	Characterise spatial spread or spatial heterogeneity	Compartmental	Susceptible, exposed, infectious, recovered	Yes	Yes	MCMC	Incidence of cases	All	No	No	R	https://github.com/RachidMuleia/Sierra_Leone_Data/blob/master/SEIR_epidemic_model_code	Yes but not informative
Kramer 2016	100.00	Characterise spatial spread or spatial heterogeneity	Compartmental	Susceptible (admin unit), infected (admin unit)	Yes	Yes	MLE	Infected status of admin units	Early	Yes	No	R	https://datadryad.org/stash/dataset/doi:10.5061/dryad.k95j3	Yes
Ivorra 2015	42.86	Transmission	Compartmental	infected, Infectious, hospitatlised, dead, buried, recovered	No	Yes	Unclear	Cumulative incidence of cases AND deaths	All	Yes	No	MATLAB	http://www.mat.ucm.es/~ivorra/softbecodis.htm	Yes
Hart 2019	71.43	Forecast	Compartmental	Susceptible, exposed, infectious, recovered	No	Yes	Other	Cumulative incidence of cases	All	No	No	MATLAB	https://github.com/will-s-hart/EbolaVariableSymptoms	No
Funk 2019	100.00	Forecast	Compartmental	Susceptible, exposed, infectious, recovered	Yes	Yes	MCMC	Incidence of cases	All	No	No	R	https://doi.org/10.5281/zenodo.2547701	Yes
Funk 2018	100.00	Forecast	Compartmental	Susceptible, exposed, infectious (unreported and potentially reported), removed (recovered or dead)	Yes	Yes	MCMC	Simulated data	All	No	No	R	http://github.com/sbfnk/ebola_forecasting_challenge	Yes
D'Silva 2017*	71.43	Transmission	Compartmental	Susceptible, exposed, two-stage infection, funeral, recovered, dead	Yes	Yes	Unclear	Incidence of cases AND deaths		Yes	No	MATLAB	https://github.com/epimath/gravity-model-ebola	Yes but not informative
Althaus 2015 (b)	71.43	Transmission	Compartmental	Susceptible, exposed, infectious, recovered, dead	Yes	Yes	MLE	Incidence of cases	All	No	No	R	https://github.com/calthaus/Ebola and supplementary material files	Yes
Abbate 2016	85.71	Transmission	Compartmental	Susceptible, exposed, infectious, recovered, dead	Yes	Yes	MLE	Incidence of cases	Middle	No	No	R	https://doi.org/10.1371/journal.pntd.0004676.s007	Yes
Abbate 2016	85.71	Sexual transmission impact	Compartmental	Susceptible, exposed, infectious, convelescent (sexual transmission from survivors), recovered, dead	Yes	No		Simulated data	Middle	No	No	R	https://doi.org/10.1371/journal.pntd.0004676.s008	Yes
Vaughan 2019	57.14	Methodological development	Other or combination	Susceptible, infectious, recovered	Yes	Yes	MCMC	Sequencing data	All	No	No	R	https://github.com/tgvaughan/ParticleFilterResults	Yes
Santermans 2016	100.00	Transmission	Other or combination	Susceptible, exposed, infectious who receover, recovered, infectious who die, dead infectious, dead not infectious	Yes	Yes	MCMC	Incidence of cases AND deaths	All	Yes	No	R	https://zenodo.org/records/33126	Yes but not informative
Lau 2017	85.71	Methodological development	Other or combination	Susceptible, exposed, infectious, recovered	Yes	Yes	MCMC	Individual times of clinical events (e.g. symptom onset, death)	Middle	Yes	No	R, C++	https://github.com/msylau/A-mechanistic-spatio-temporal-framework-for-modelling-individual-to-individual-transmission-Ebola	Yes but not informative
De Maio 2018	100.00	Transmission	Other or combination	Not compartmental	Yes	Yes	MCMC	Sequencing data	Early	No	No	BEAST2	https://doi.org/10.1371/journal.pcbi.1006117.s002	No

5 Pathogen Epidemiology Review Group (PERG) membership

Table S28: Pathogen Epidemiology Review Group (PERG) membership

First Name	Surname	Affiliation
Aaron	Morris	University of Oxford
Alpha	Forna	University of Georgia
Amy	Dighe	Johns Hopkins
Anna-Maria	Hartner	Imperial College London
Anna	Vicco	Imperial College London
Anne	Cori	Imperial College London
Arran	Hamlet	Imperial College London
Ben	Lambert	University of Oxford
Bethan	Daniels	Imperial College London
Charlie	Whittaker	Imperial College London
Christian	Morgenstern	Imperial College London
Cosmo	Santoni	Imperial College London
Cyril	Geismar	Imperial College London
Dariya	Nikitin	Imperial College London
David	Jorgensen	Imperial College London
Dominic	Dee	Imperial College London
Ed	Knock	Imperial College London
Gina	Cuomo-Dannenburg	Imperial College London
Hayley	Thompson	PATH
Ilaria	Dorigatti	Imperial College London
Isobel	Routledge	UCSF
Janetta	Skarp	Imperial College London
Joseph	Hicks	Imperial College London
Juliette	Unwin	University of Bristol
Kanchan	Parchani	Imperial College London
Keiran	Drake	Imperial College London
Keith	Fraser	Imperial College London
Kelly	Charniga	Imperial College London
Kelly	McCain	Imperial College London
Lily	Geidelberg	Imperial College London
Lorenzo	Cattarino	UKHSA
Mara	Kont	Imperial College London
Marc	Baguelin	Imperial College London
Natsuko	Imai-Eaton	Imperial College London (Wellcome Trust)
Nima	Moghaddas	Imperial College London
Pablo	Guzman	Imperial College London
Patrick	Doohan	Imperial College London
Paula	Christen	Imperial College London
Rebecca	Nash	Imperial College London
Richard	Shepherd	Imperial College London
Rob	Johnson	Imperial College London
Ruth	McCabe	Imperial College London
Sabine	van Elsland	Imperial College London
Sangeeta	Bhatia	UK Health Security Agency

Sequoia	Leuba	Imperial College London
Shazia	Ruybal-Pesantez	Imperial College London
Sreejith	Radhakrishnan	University of Glasgow
Thomas	Rawson	Imperial College London
Tristan	Naidoo	Imperial College London
Zulma	Cucunuba Perez	Pontificia Universidad Javeriana
Jack	Wardle	Imperial College London

References

1. Agency UHS. Ebola: overview, history, origins and transmission. accessed on 15/03/24. 2023. Available from: https://www.gov.uk/government/publications/ebola-origins-reservoirs-transmission-and-guidelines/ebola-overview-history-origins-and-transmission
2. Van Kerkhove MD, Bento AI, Mills HL, Ferguson NM, and Donnelly CA. A review of epidemiological parameters from Ebola outbreaks to inform early public health decision-making. Scientific data 2015; 2:1–10
3. Rojek A, Salam A, Ragotte R, Liddiard E, Elhussain A, Carlqvist A, Butler M, Kayem N, Castle L, Odondi L, Stepniewska K, and Horby P. A systematic review and meta-analysis of patient data from the West Africa (2013–16) Ebola virus disease epidemic. Clinical Microbiology and Infection 2019; 25:1307–14. DOI: https://doi.org/10.1016/j.cmi.2019.06.032. Available from: https://www.sciencedirect.com/science/article/pii/S1198743X19303878
4. Kawuki J, Musa T, and Yu X. Impact of recurrent outbreaks of Ebola virus disease in Africa: a meta-analysis of case fatality rates. Public Health 2021; 195:89–97. DOI: https://doi.org/10.1016/j.puhe.2021.03.027. Available from: https://www.sciencedirect.com/science/article/pii/S0033350621001396
5. Nyakarahuka L, Kankya C, Krontveit R, Mayer B, Mwiine FN, Lutwama J, and Skjerve E. How severe and prevalent are Ebola and Marburg viruses? A systematic review and meta-analysis of the case fatality rates and seroprevalence. BMC infectious diseases 2016; 16:1–14
6. Belhadi D, El Baied M, Mulier G, Malvy D, Mentré F, and Laouénan C. The number of cases, mortality and treatments of viral hemorrhagic fevers: A systematic review. PLOS Neglected Tropical Diseases 2022 Oct; 16:1–25. DOI: 10.1371/journal.pntd.0010889. Available from: https://doi.org/10.1371/journal.pntd.0010889
7. Brainard J, Hooper L, Pond K, Edmunds K, and Hunter PR. Risk factors for transmission of Ebola or Marburg virus disease: a systematic review and meta-analysis. International Journal of Epidemiology 2015 Nov; 45:102–16. DOI: 10.1093/ije/dyv307. eprint: https://academic.oup.com/ije/article-pdf/45/1/102/24170592/dyv307.pdf. Available from: https://doi.org/10.1093/ije/dyv307
8. Selvaraj SA, Lee KE, Harrell M, Ivanov I, and Allegranzi B. Infection Rates and Risk Factors for Infection Among Health Workers During Ebola and Marburg Virus Outbreaks: A Systematic Review. The Journal of Infectious Diseases 2018 Sep; 218:S679–S689. DOI: 10.1093/infdis/jiy435. eprint: https://academic.oup.com/jid/article-pdf/218/suppl_5/S679/26689050/jiy435.pdf. Available from: https://doi.org/10.1093/infdis/jiy435
9. Velásquez GE, Aibana O, Ling EJ, Diakite I, Mooring EQ, and Murray MB. Time From Infection to Disease and Infectiousness for Ebola Virus Disease, a Systematic Review. Clinical Infectious Diseases 2015 Jun; 61:1135–40. DOI: 10.1093/cid/civ531. eprint: https://academic.oup.com/cid/article-pdf/61/7/1135/17352801/civ531.pdf. Available from: https://doi.org/10.1093/cid/civ531

10. Dean NE, Halloran ME, Yang Y, and Longini IM. Transmissibility and Pathogenicity of Ebola Virus: A Systematic Review and Meta-analysis of Household Secondary Attack Rate and Asymptomatic Infection. Clinical Infectious Diseases 2016 Feb; 62:1277–86. DOI: 10.1093/cid/ciw114. eprint: https://academic.oup.com/cid/article-pdf/62/10/1277/16788110/ciw114.pdf. Available from: https://doi.org/10.1093/cid/ciw114

11. Bower H and Glynn JR. A systematic review and meta-analysis of seroprevalence surveys of ebolavirus infection. Scientific data 2017; 4:1–9

12. WONG ZSY, BUI CM, CHUGHTAI AA, and MACINTYRE CR. A systematic review of early modelling studies of Ebola virus disease in West Africa. Epidemiology and Infection 2017; 145:1069–94. DOI: 10.1017/S0950268817000164

13. Suliman Jamiel M. Abdalla FC and Govinder KS. A systematic review of mathematical models of the Ebola virus disease. International Journal of Modelling and Simulation 2022; 42:814–30. DOI: 10.1080/02286203.2021.1983745. eprint: https://doi.org/10.1080/02286203.2021.1983745. Available from: https://doi.org/10.1080/02286203.2021.1983745

14. Balduzzi S, Rücker G, and Schwarzer G. How to perform a meta-analysis with R: a practical tutorial. BMJ Ment Health 2019; 22:153–60. DOI: 10.1136/ebmental-2019-300117. eprint: https://mentalhealth.bmj.com/content/22/4/153.full.pdf. Available from: https://mentalhealth.bmj.com/content/22/4/153

15. Higgins JP and Green S. Cochrane Handbook for Systematic Reviews of Interventions. accessed on 15/03/24. 2011. DOI: https://handbook-5-1.cochrane.org/chapter_7/7_7_3_2_obtaining_standard_deviations_from_standard_errors_and.htm

16. Cai S, Zhou J, and Pan J. Estimating the sample mean and standard deviation from order statistics and sample size in meta-analysis. Statistical Methods in Medical Research 2021; 30. PMID: 34668458:2701–19. DOI: 10.1177/09622802211047348. eprint: https://doi.org/10.1177/09622802211047348. Available from: https://doi.org/10.1177/09622802211047348
